# Case report: Ofatumumab treatment in anti-DPPX autoimmune encephalitis

**DOI:** 10.3389/fimmu.2024.1320608

**Published:** 2024-06-27

**Authors:** Peicai Fu, Zhenqiong Hu, Guopeng Zhang, Zhijun Li

**Affiliations:** ^1^ Department of Neurology, Tongji Hospital, Tongji Medical College, Huazhong University of Science and Technology, Wuhan, Hubei, China; ^2^ Department of Neurology, Hubei Aerospace Hospital, Xiaogan, Hubei, China; ^3^ Department of Nuclear Medicine, Tongji Hospital, Tongji Medical College, Huazhong University of Science and Technology, Wuhan, Hubei, China

**Keywords:** anti-dipeptidyl peptidase-like protein 6 (anti-DPPX), autoimmune encephalitis, neuropsychiatric symptoms, ofatumumab, immunotherapy

## Abstract

Dipeptidyl peptidase-like protein 6 (DPPX) antibody encephalitis is a rare autoimmune encephalitis. Diagnosis and treatment of DPPX remain challenging, particularly in patients with refractory disease. Herein, we report the first case of anti-DPPX encephalitis treated with ofatumumab. The patient had a chronic insidious onset and predominantly presented with severe neuropsychiatric symptoms and the typical triad of symptoms (weight loss, central nervous system hyperexcitability, and cognitive dysfunction). Positive anti-DPPX antibodies in the serum (1:1,000) and cerebrospinal fluid (CSF) (1:100) were detected at the disease peak. The patient was unresponsive to four types of standard immunotherapies (intravenous globulin, plasma exchange, steroids, and tacrolimus), resulting in a treatment switch to ofatumumab. After five doses of injection and 12 months of follow-up, the patient improved well, with only a mild cognitive deficit.

## 1 Introduction

Anti-dipeptidyl peptidase-like protein 6 (DPPX) encephalitis, caused by cell surface autoantigens to DPPX, is a rare form of autoimmune encephalitis (AE) characterized by various symptoms, including memory loss, sleep disturbance, psychiatric symptoms, ataxia, seizures, central hyperexcitability, tremors, myoclonus, diarrhea, and weight loss, among others (1). DPPX is the cell surface regulatory subunit of the Kv4.2 potassium channel, which is located in the neuronal somata and dendrites in the brain and in the myenteric plexus of the small intestine and is involved in the attenuation of backpropagation of action potentials and somato-dendritic signal integration (2, 3). As first described by Boronat et al. in 2013 (4), DPPX expression in multiple regions, including the nervous system and myenteric plexus, explains the multifocal clinical features of DPPX encephalitis, including the observed cognitive dysfunction and neuropsychiatric and gastrointestinal symptoms. Unlike other paraneoplastic AEs, the etiology of anti-DPPX encephalitis is not typically a paraneoplastic process; however, a few cases have been reported to be associated with lymphoproliferative neoplasms (5). AEs are highly reversible if diagnosed and treated promptly. Immunotherapy is widely used for AEs and has been proven to be beneficial for most patients with anti-DPPX encephalitis. Regrettably, a recent study on DPPX encephalitis with a median follow-up of 46 months revealed that complete recovery is unlikely and long-term cognitive deficits persist even after receiving immunotherapy (6). Moreover, some patients fail to respond to first-line treatment.

B-cell-depleting monoclonal antibodies (mAbs) directed against CD20 have been verified as effective therapies for many autoimmune diseases, including AEs associated with different autoantibodies (7–9). Ofatumumab, a recombinant human monoclonal immunoglobulin G1 antibody, is an acknowledged second-generation anti-CD20 mAb that binds to B cells expressing human CD20 and functions by depleting B lymphocytes. Ofatumumab has been approved for the treatment of relapsing-remitting multiple sclerosis (MS). Moreover, ofatumumab has shown promising results in patients with antineutrophil cytoplasmic antibody-associated vasculitis, rheumatoid arthritis, systemic lupus erythematosus, etc. (9).

Ofatumumab has recently been reported as an effective treatment in some cases of AEs (10, 11). However, the efficacy of ofatumumab for anti-DPPX encephalitis remains uncertain. Herein, we report a patient with anti-DPPX encephalitis who showed significant improvements after receiving five injections of ofatumumab during a nearly 12-month follow-up period.

## 2 Case report

A 51-year-old male patient began to experience excessive anxiety about his health in June 2021, accompanied by weight loss, reduced interest, reduced speech, and dizziness. He further experienced a reduced appetite, reportedly triggered by a strange smell while eating. He had previously undergone surgery for a benign polyp of the colon 2 years prior, but otherwise had no remarkable medical or family history of inherited diseases. He was diagnosed with depression at a local neuropsychiatric ward and was prescribed antidepressant treatment without improvement. The patient’s symptoms worsened gradually. In March 2022, he developed cognitive impairment, temporal and spatial disorientation, speech disturbances, irritability, and mild hand tremors. Shortly thereafter, he underwent brain magnetic resonance imaging (MRI), revealing no abnormalities.

The patient’s cognitive impairment worsened 5 months later. He occasionally could not recognize his wife and experienced intermittent confusion and hallucinations. He further progressively developed sphincter dysfunction, limb rigidity, and resting and postural tremors in the jaw and limbs, which were relieved by sleep. He was admitted to the local hospital, where a second brain MRI still revealed no abnormalities, while an electroencephalogram (EEG) showed diffuse slow waves. Anti-DPPX antibodies were detected in the serum (1:100) and cerebrospinal fluid (CSF) (1:100). He was diagnosed with anti-DPPX encephalitis, which was initially treated by intravenous methylprednisolone impulse (1 g for 5 days, followed by oral tapering) and intravenous immunoglobulin (0.4 g/kg for 5 days), with no obvious improvement. Ultimately, he was transferred to our emergency room (Tongji Hospital, Wuhan, China) 1 month later, after his condition had further deteriorated to include a disturbance of consciousness, and he was subsequently admitted to the intensive care unit.

On neurological examination, the patient showed impaired consciousness, and he could not cooperate with the physical examination. Persistent tremors were observed in the jaw and extremities, accompanied by intermittent myoclonus. His muscle tone was rigid, while deep tendon reflexes were normal. No pathological signs were observed. His neck was stiff, and the Kernig sign was negative. He experienced a total weight loss of around 25–30 kg over a period of one and a half years.

**Figure 1:**
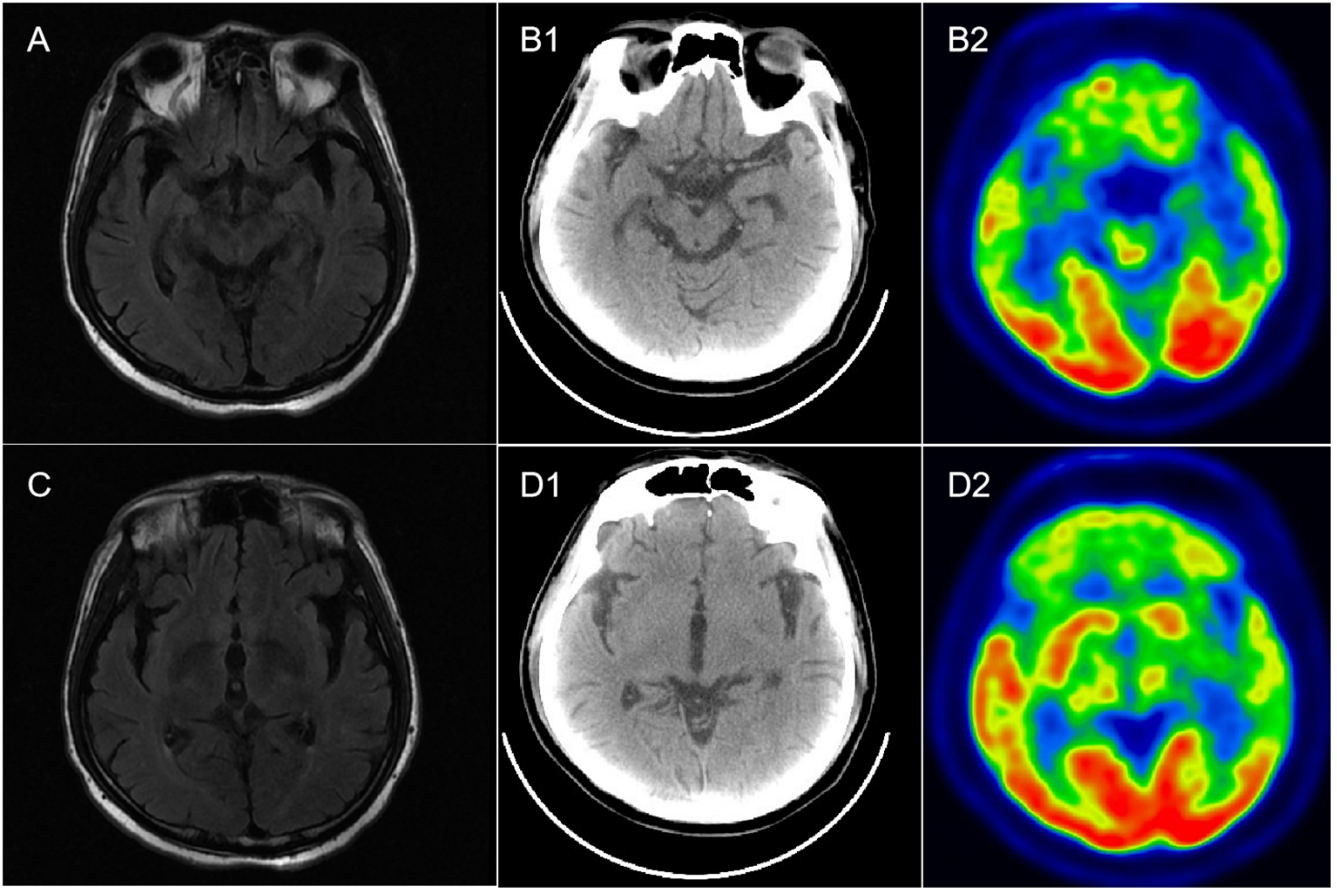
Brain MR and 18F-FDG PET-CT imaging in a patient with anti-DPPX encephalitis. **(A**, **C)** Mild atrophy of the hippocampus and temporal lobe in T2-flair imaging. **(B1**, **B2)** Normal metabolism in both hippocampus in PET-CT. **(D1**, **D2)** Hypometabolism in the bilateral frontal lobes, parietal lobes, and temporal lobes, especially the left side.

**Figure 2:**
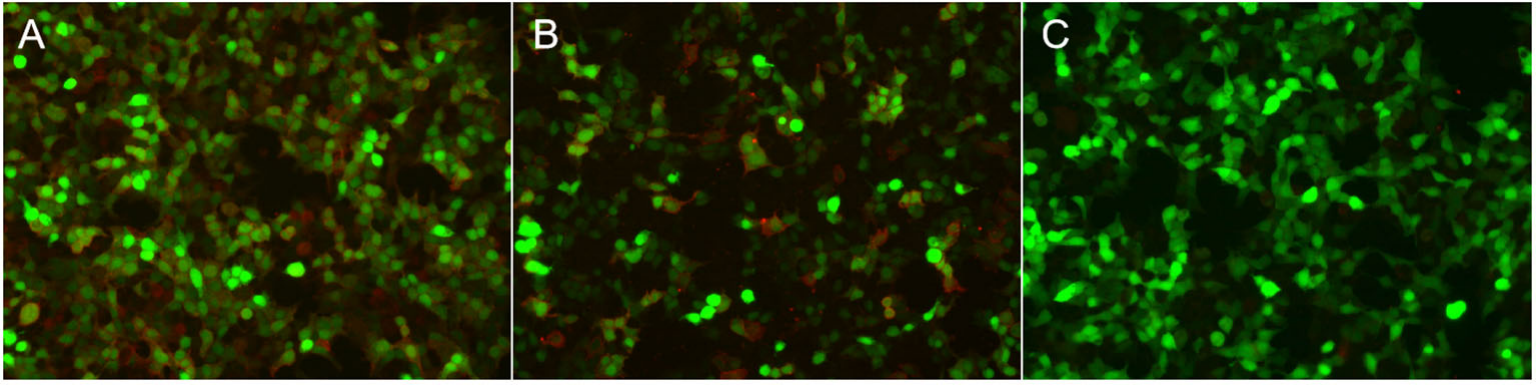
Immunofluorescence in serum and CSF in patients with anti-DPPX encephalitis assessed by cell-based assay (CBA). Positive reaction with transfected HEK 293 cells expressing DPPX after incubation with the patient’s serum **(A)** (titer: 1:1,000) and with CSF **(B)** (titer: 1:100). **(C)** Negative control.

Routine serological investigations (including for M protein, syphilis, hepatitis B, and HIV) and other tests for tumors and systemic autoimmune diseases were all negative. The results of nerve conduction studies, chest and abdominal computed tomography (CT), and bone marrow biopsy were also normal. A third MRI and enhanced scans of the brain revealed non-specific changes with atrophy (**Figure 1**), while the spinal cord MRI was unremarkable. EEG showed extensive slowing of the background frequency. CSF analysis further revealed a slightly elevated protein level (0.602 g/L), with normal open pressure and white blood cell count. Oligoclonal bands were not observed. Whole-body 18FDG-PET/CT revealed markedly decreased metabolic activity in the bilateral frontal, parietal, and temporal lobes (particularly on the left side). No related tumors were found on 18FDG-PET/CT. Subsequent testing for DPPX antibodies using cell-based assays revealed positivity in both the serum (1:1000) and CSF (1:100) (**Figure 2**). Tests for other autoimmune and paraneoplastic antibodies were negative. The diagnosis of anti-DDPX encephalitis was thus confirmed.

After five rounds of plasma exchange with intravenous methylprednisolone (80 mg), the patient recovered consciousness. However, he still presented with speech disturbances, impulsiveness, aggressive behavior, and uncontrolled tremors in the limbs and jaw. Scratching marks of neurogenic pruritus were also observed. Neurocognitive testing revealed a Mini-Mental State Examination (MMSE) score of 13/30 and a Montreal Cognitive Assessment (MOCA) score of 13/30, with a Modified Rankin Scale (mRS) score of 4. Tapering doses of oral steroids and tacrolimus (TAC) were administered concurrently. Despite sufficient immunosuppression with oral prednisone (40 mg/day) and TAC (1.5 mg, twice daily) for at least one month, his symptoms alleviated only very slowly, with cognitive impairment and tremors in his extremities being particularly refractory. Therefore, treatment with ofatumumab, a B-cell-depleting agent, was suggested. Prior to the use of ofatumumab, the patient was assessed to ensure the absence of a pulmonary infection or any other infection. For personal and economic reasons, the protocol for the injection of ofatumumab was individualized, with injections scheduled to be resumed only when the CD19^+^ B-cell count exceeded 10/µL. During the 12-month follow-up period, he received five injections of ofatumumab.

## 3 Outcomes

**Figure 3:**
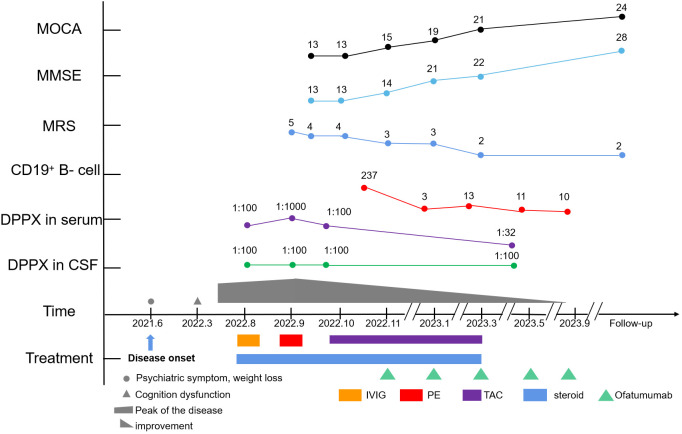
Timeline of the clinical course, changes in the MMSE, MoCA, mRS, CD19^+^ B-cell count, and different treatment regimens of the case.

The patient showed improvements in neuropsychiatric symptoms shortly after the first injection. The percentage of CD19^+^ B cells decreased to 0.08% following the first injection. At the 2-month follow-up visit, the patient showed dramatic improvements in speech disturbances, disorientation, and behavioral disorders, except for tremors in both limbs and memory deficits. At the 3-month follow-up, oral prednisone and tacrolimus were discontinued after achieving symptom remission. A fourth brain MRI scan revealed nonspecific changes. Tremors were almost completely alleviated after 5 months (**Figure 3**). The patient tolerated the treatment well.

At the final follow-up, the patient was stable but still experienced mild cognitive impairment with occasional tremors. No significant adverse effects, allergies, or relapses were observed. Serum IgM levels were within the normal range (0.66 g/L). The mRS score was 2, the MMSE score was 28/30, and the MOCA score was 24/30. During the follow-up period, the patient was able to take care of himself in daily life and showed no signs of infection. Even during the Omicron disease outbreak, the patient successfully navigated treatment and daily life. The patient and his family were satisfied with treatment outcomes, and ofatumumab treatment has therefore been maintained.

## 4 Discussion

In this report, we presented the efficacy and safety of ofatumumab in a rare case of refractory anti-DPPX encephalitis. This case report demonstrates that ofatumumab is a promising treatment option for refractory AEs, including anti-DPPX encephalitis.

To date, only a few cases of anti-DPPX encephalitis have been reported, with significant heterogeneity in clinical and imaging features. In our case, the patient had an insidious onset with acute progression of the neurological disorder, reaching a disease peak at approximately 5 months. The patient primarily presented with neuropsychiatric symptoms; however, other cortical dysfunctions were also prominent, including confusion, cognitive dysfunction, memory deficits, and speech dysfunction. Hyperexcitability symptoms of the CNS, such as tremors, persisted throughout almost the entirety of the disease course, while limb rigidity and occasional myoclonus were observed during the disease peak. However, other symptoms of hyperexcitability, including exaggerated startle, hyperekplexia, and seizures, were not observed (12). Moreover, the patient manifested other multiregional symptoms such as gastrointestinal symptoms, sphincter dysfunction, weight loss, and neurogenic pruritus.

Although a variety of clinical presentations and extensive dysfunction were observed, classical radiological features of AEs were absent, which was inconsistent with our previous report (1). During the entire disease course, four brain MRI scans were performed, with none indicating any obvious abnormal signals except for diffuse atrophy. “Encephalitis with normal MRI” has previously been classified as a clinical subtype of AEs (13). However, the spectrum of “encephalitis with normal MRI” ranges significantly. In some recent cases of anti-DPPX encephalitis, normal brain MRI findings have been displayed (14, 15). Moreover, normal MRI was also observed in another clinical variant of anti-DPPX encephalitis, named “progressive encephalomyelitis with rigidity and myoclonus (PERM)” (12), which is characterized by hyperexcitability. In addition, in a recent report of three cases with “encephalitis with normal MRI”, patients were associated with thymoma as well as a concurrent antibody against anti-amino-3-hydroxy-5-methyl-4-isoxazolepropionic acid receptor (AMPA) (13). These cases indicate that traditional MRI is not always sufficiently sensitive to evaluate intracranial lesions in AEs. PET-CT, as a supplementary examination in the current case, revealed hypometabolism in the bilateral cortical regions (particularly on the left side) when the brain MRI was normal. Similar hypometabolism was also observed in other cases of anti-DPPX encephalitis (16, 17), indicating that PET-CT/MRI may be helpful both to screen tumors and to overcome the limitations of traditional MRI.

As previously reported, most patients with anti-DPPX encephalitis benefit from traditional immunotherapies (1). However, if patients are unresponsive to first-line therapy, novel and suitable treatments should be considered. In the present report, the patient showed resistance to four types of standard immunotherapies (IVIG, PE, steroids, and TAC), which may have resulted from a high titer of anti-DPPX antibody in both the serum and CSF. High DPPX antibody titers in the CSF indicate intrathecal synthesis, which challenges the routine treatment of variant PERM (12). Therefore, in our case, the patient was switched to a safer and more effective immunosuppressant, ofatumumab.

Fortunately, our refractory patient showed an unexpected response to subcutaneous ofatumumab treatment, with this treatment achieving rapid improvements in neuropsychiatric symptoms within 1 month of treatment. Subsequently, the other symptoms were gradually alleviated. The most refractory sign, tremor, was relieved approximately 5 months after treatment with ofatumumab. The patient has been followed up for nearly 12 months, with no relapse being observed. RTX, a routine B-cell-depleting agent, is extensively used in the treatment of autoimmune diseases (8). RTX administration must be followed by monitoring in a hospital, which is inconvenient for most patients. In addition, RTX is limited by side effects, such as acute allergic reactions and a higher risk of infection. Compared to RTX, ofatumumab is a novel, fully humanized anti-CD20 mAb with a higher efficiency in B-cell depletion. Another advantage of ofatumumab is that it can be self-administered at home via subcutaneous injection. Notably, the patient presented herein tolerated ofatumumab well. However, further research is warranted to explore the long-term efficacy and safety of ofatumumab in the treatment of anti-DPPX encephalitis, even in AEs associated with different antibodies.

In summary, this was a relatively rare case and the first report of ofatumumab treatment for anti-DPPX encephalitis. This experience suggests that timely ofatumumab treatment may be an effective strategy in patients with AEs who do not benefit from conventional immunotherapy, especially in refractory cases.

## Data availability statement

Further inquiries can be directed to the corresponding author. Requests to access these datasets should be directed to ZL, zjlhuazhong@163.com.

## Ethics statement

Written informed consent was obtained from the individual(s) for the publication of any potentially identifiable images or data included in this article.

## Author contributions

PF: Conceptualization, Data curation, Investigation, Writing – original draft, Writing – review & editing. ZH: Data curation, Writing – original draft. GZ: Data curation, Writing – original draft. ZL: Conceptualization, Data curation, Writing – original draft, Writing – review & editing.
