# Supplementary material for: Case report: Ofatumumab treatment in anti-DPPX autoimmune encephalitis
Source: Front Immunol. 2024 Jun 27;15:1320608. doi: 10.3389/fimmu.2024.1320608 (PMC11240285; doi:10.3389/fimmu.2024.1320608)

LIU_SHOU_BO

Please be aware that the images contained in this electronic film may have lost information due to a lossy compression, and are not for diagnostic use.

Please note that the MPEG/AVI/MOV movies compressed with high image quality may not be played back correctly on slower machines - image skipping may occur.

Hospital:	noname
Created on:	Oct 07 2023 13:03:53
Pages:	26
JPEG Images:	26
PNG Images:	0
MPEG Movies:	0
AVI Movies:	0
MOV Movies:	0

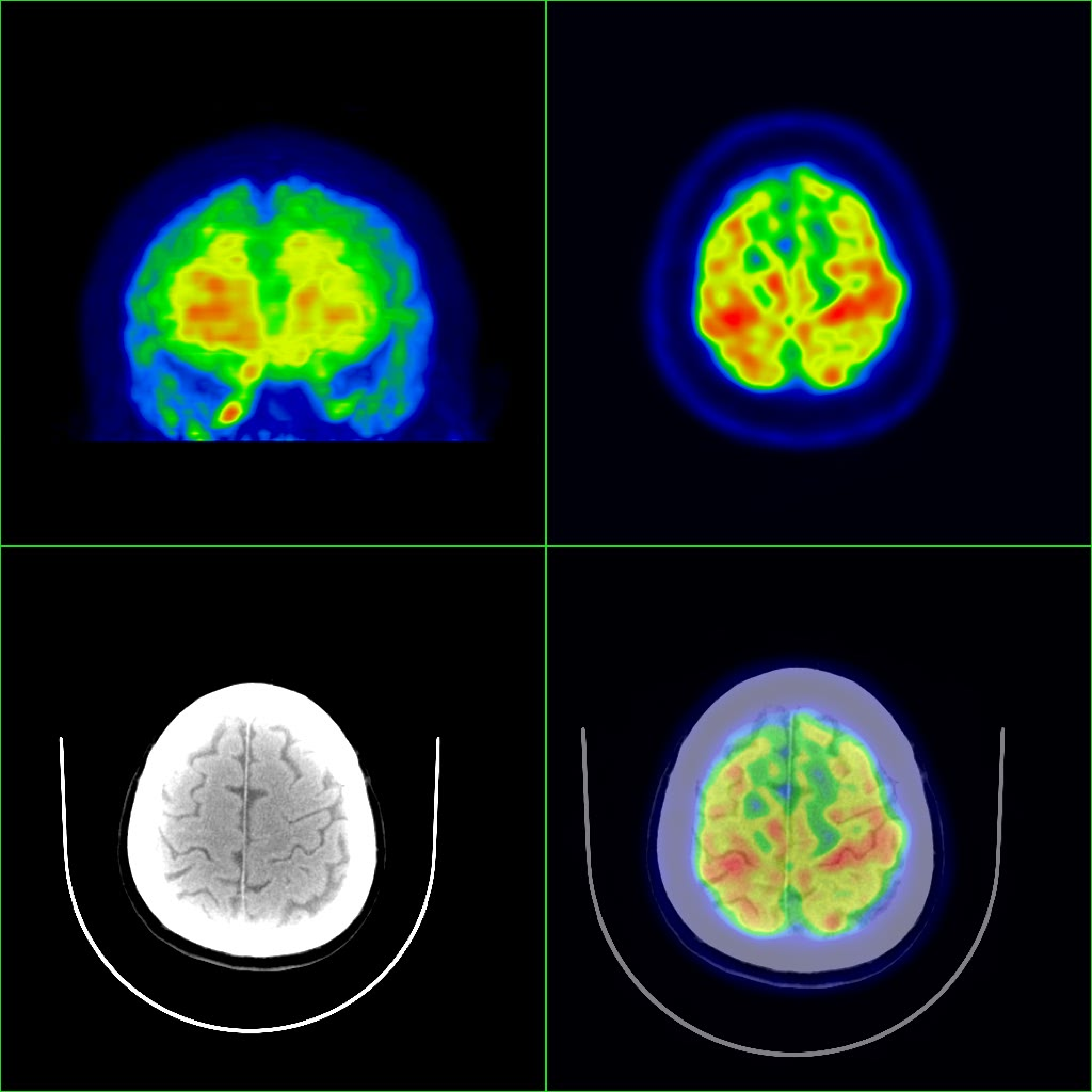

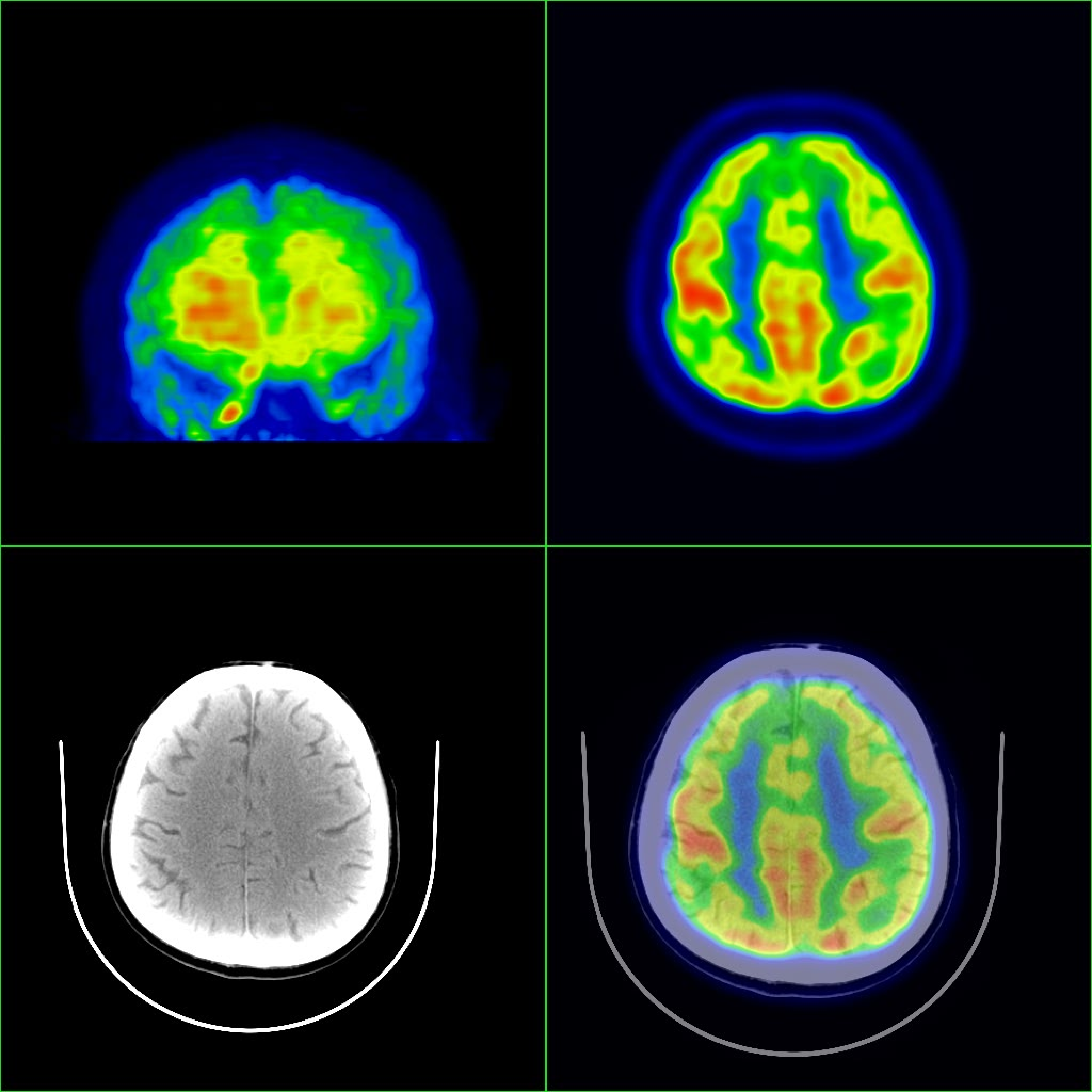

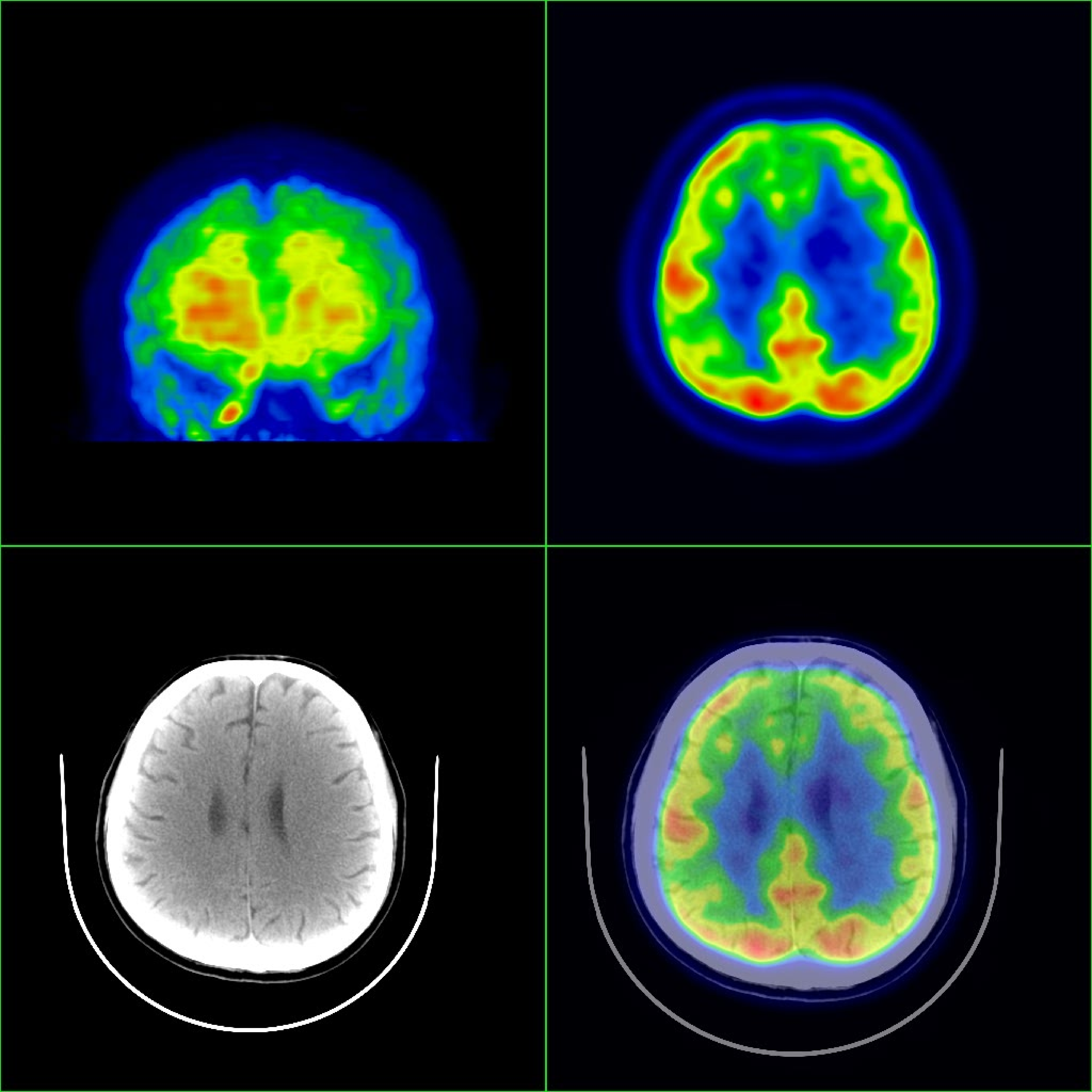

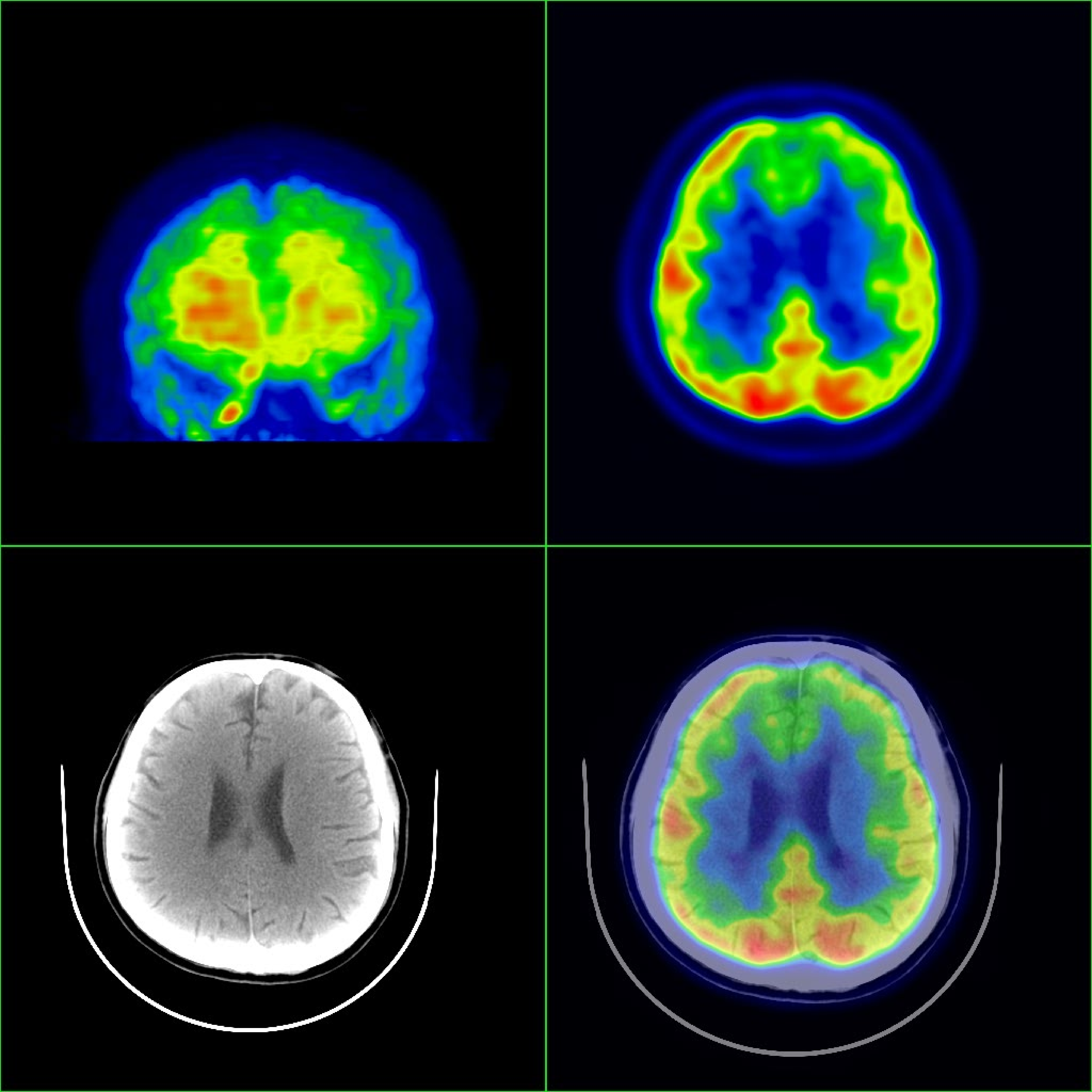

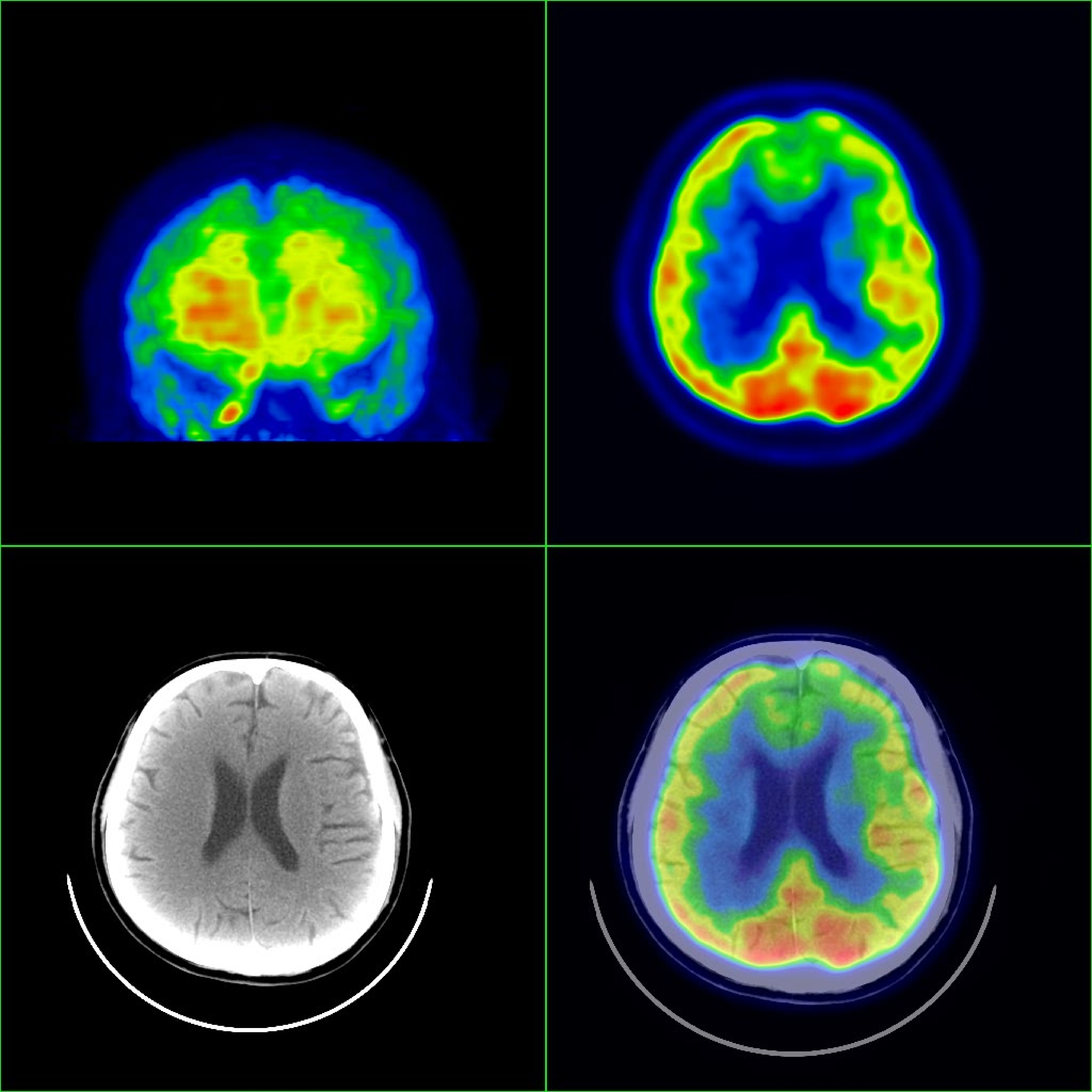

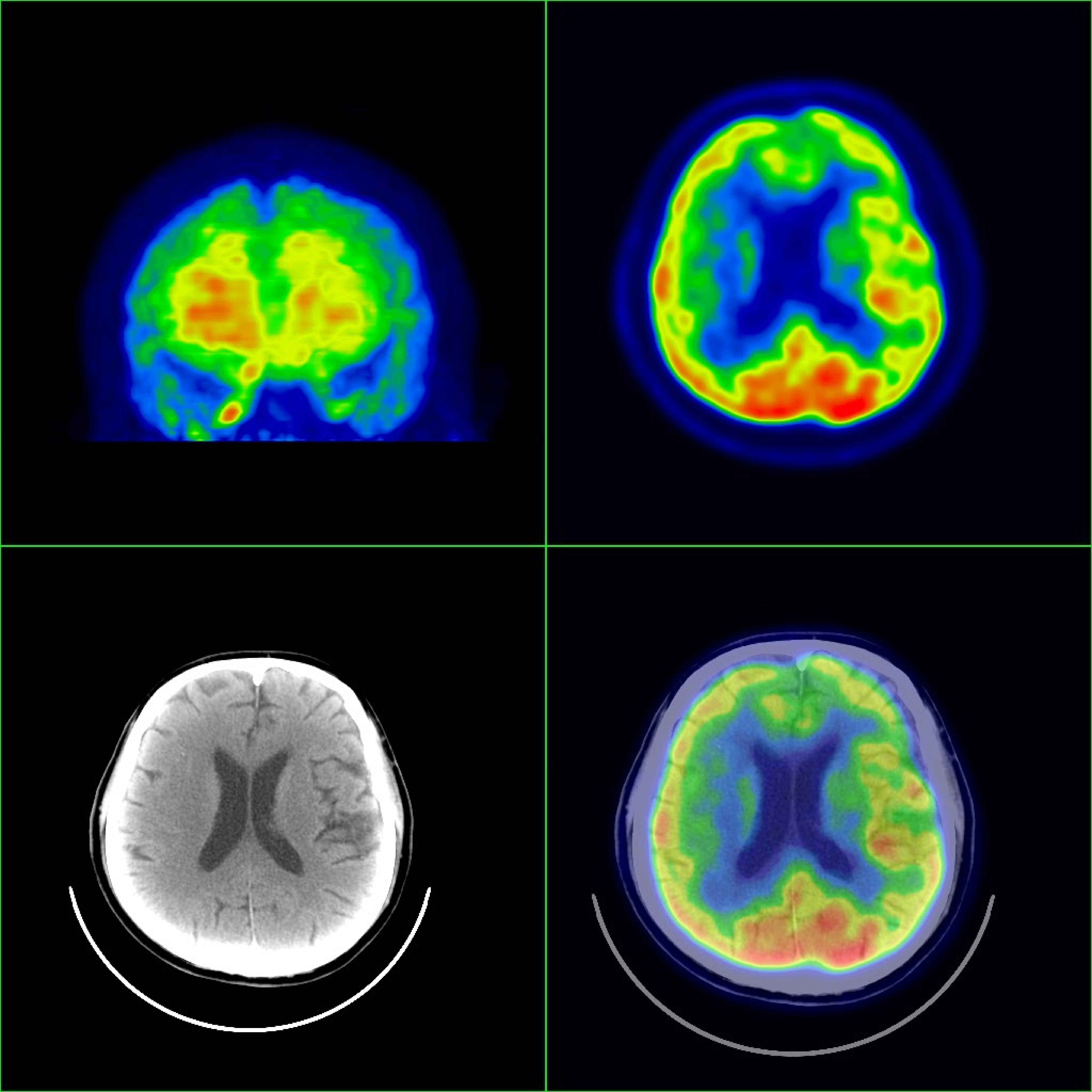

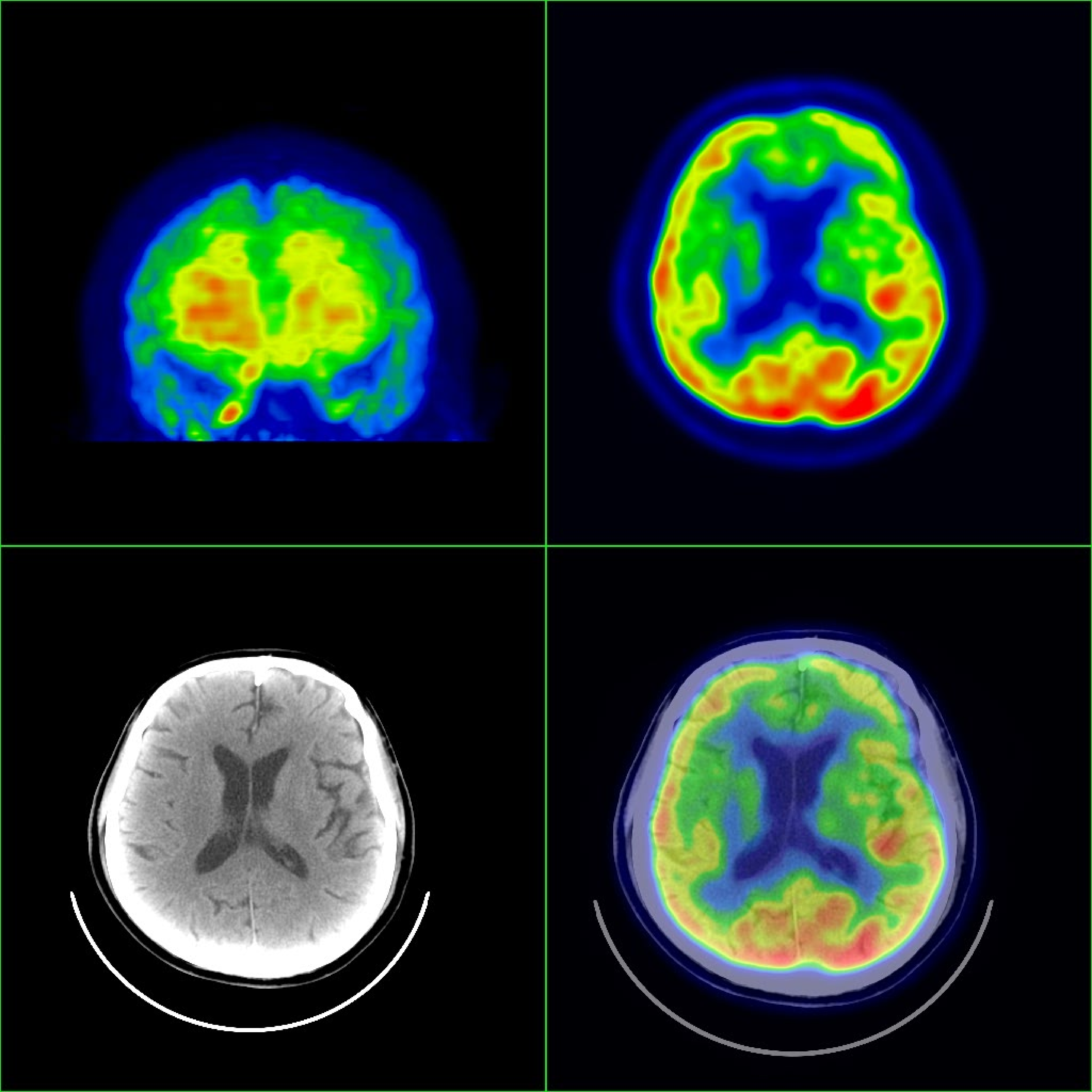

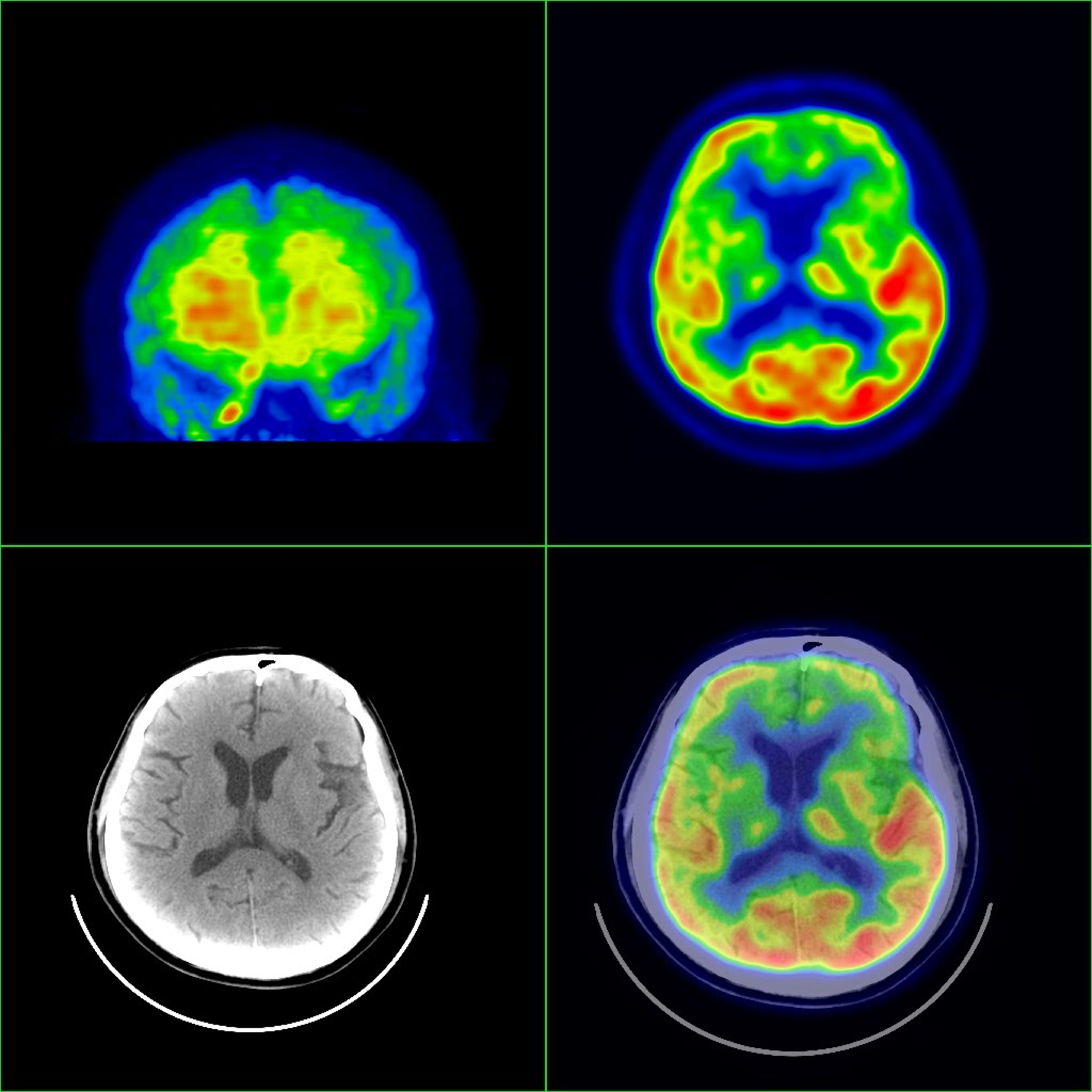

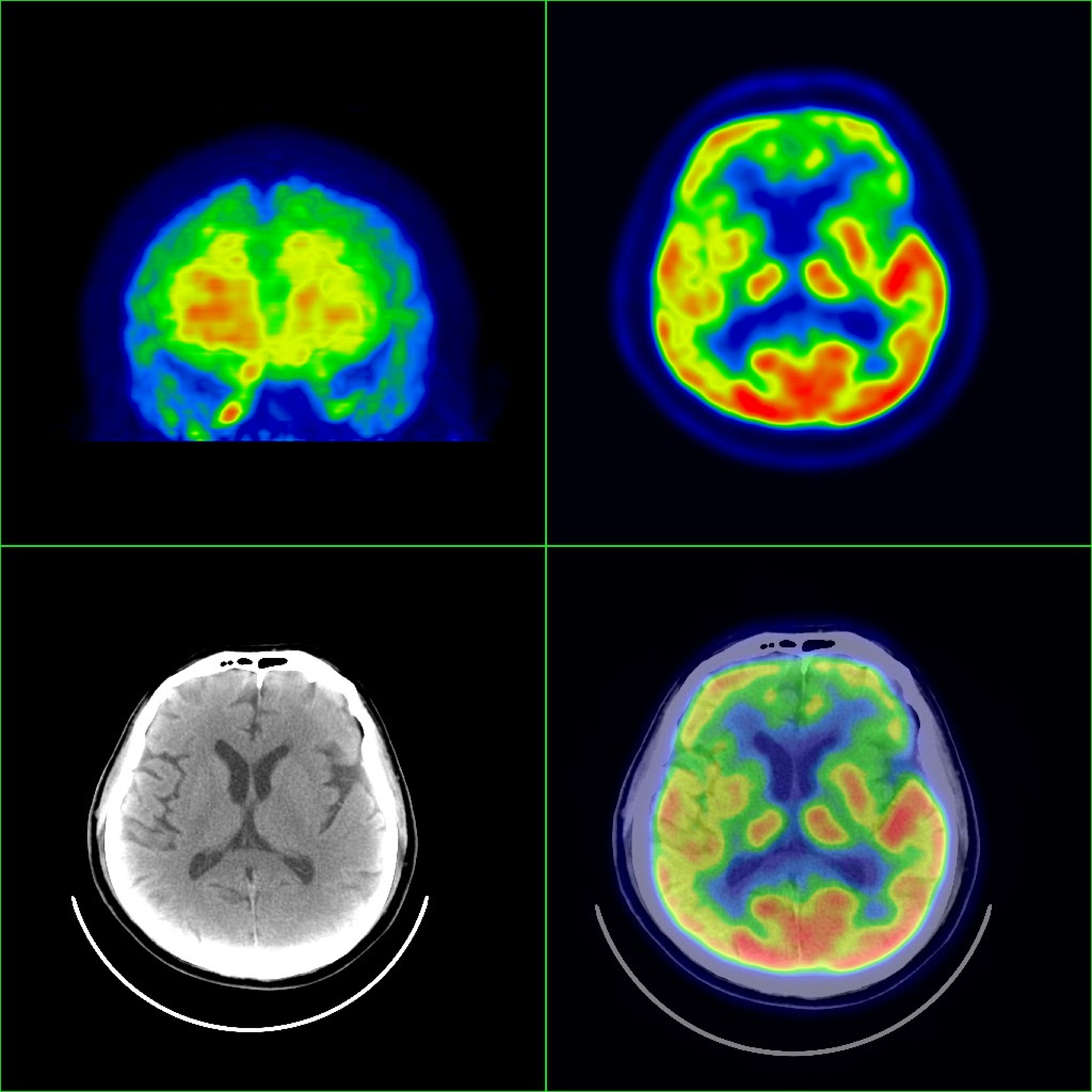

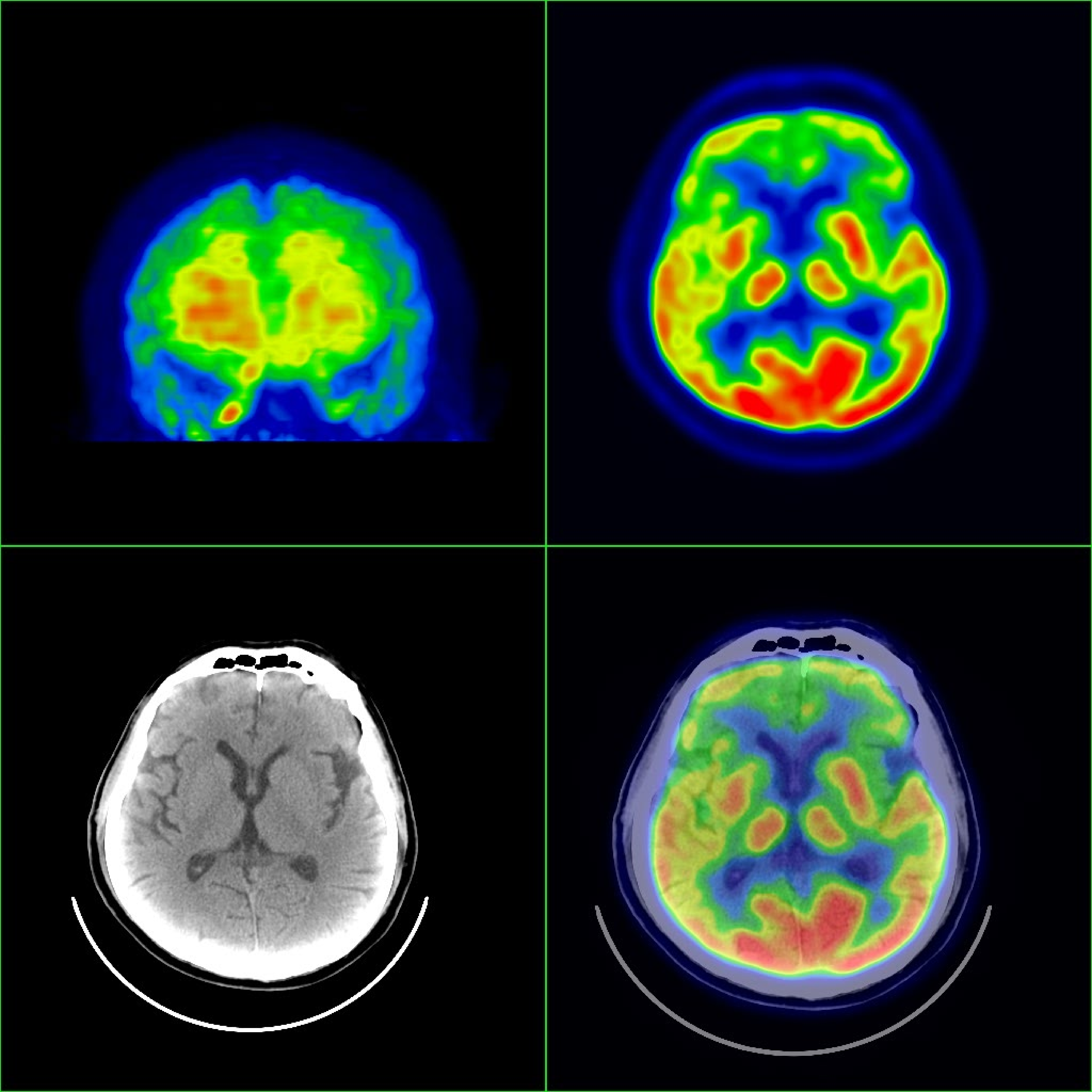

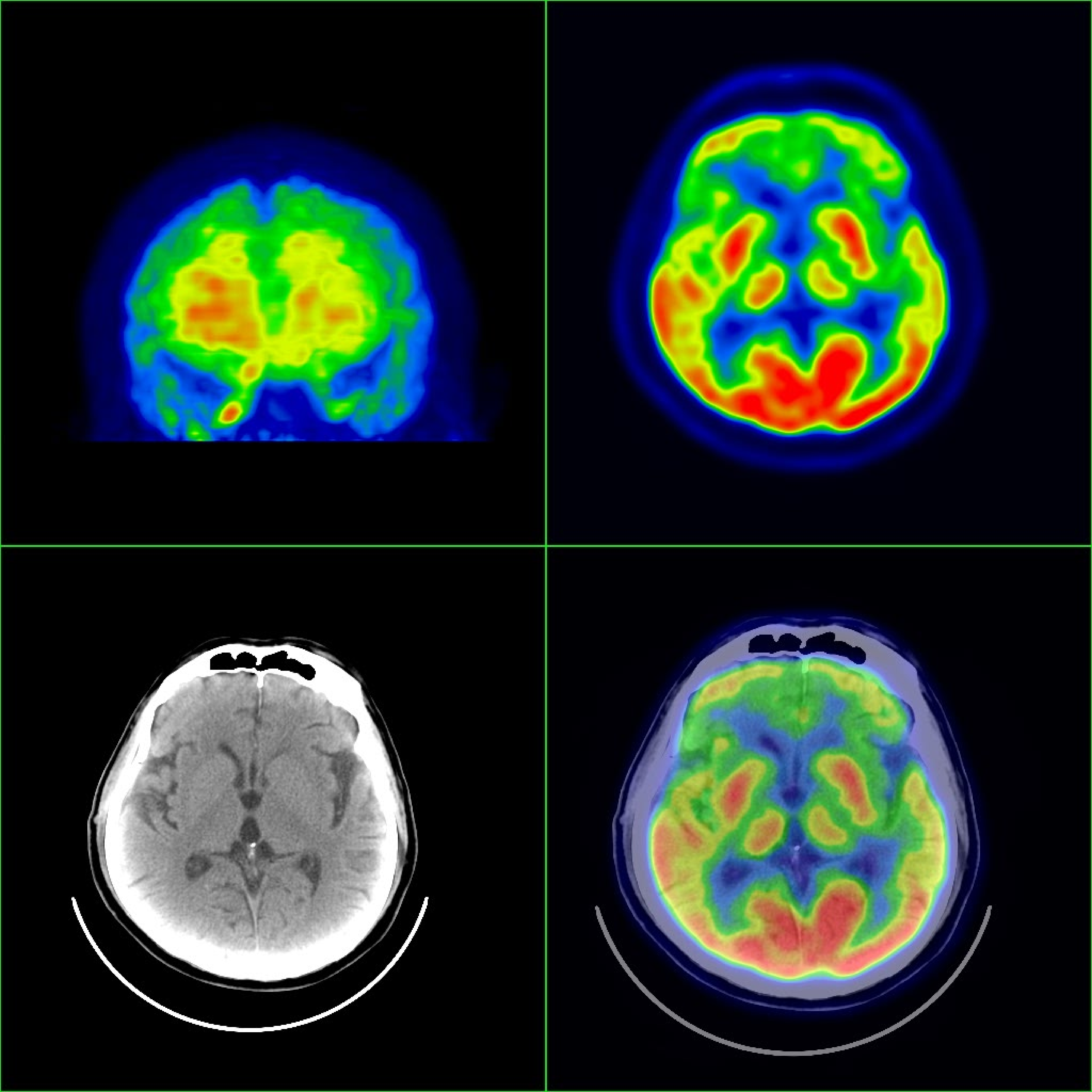

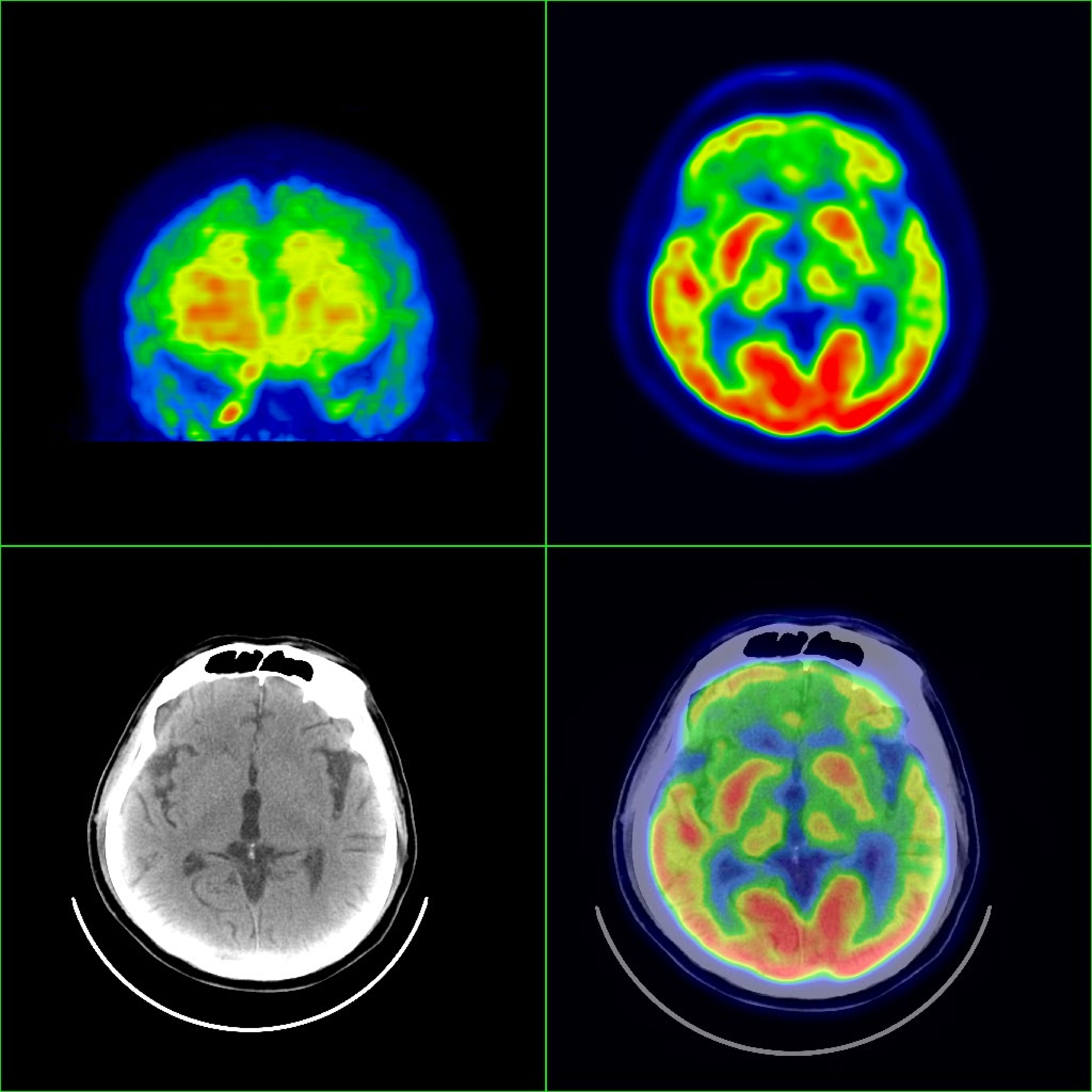

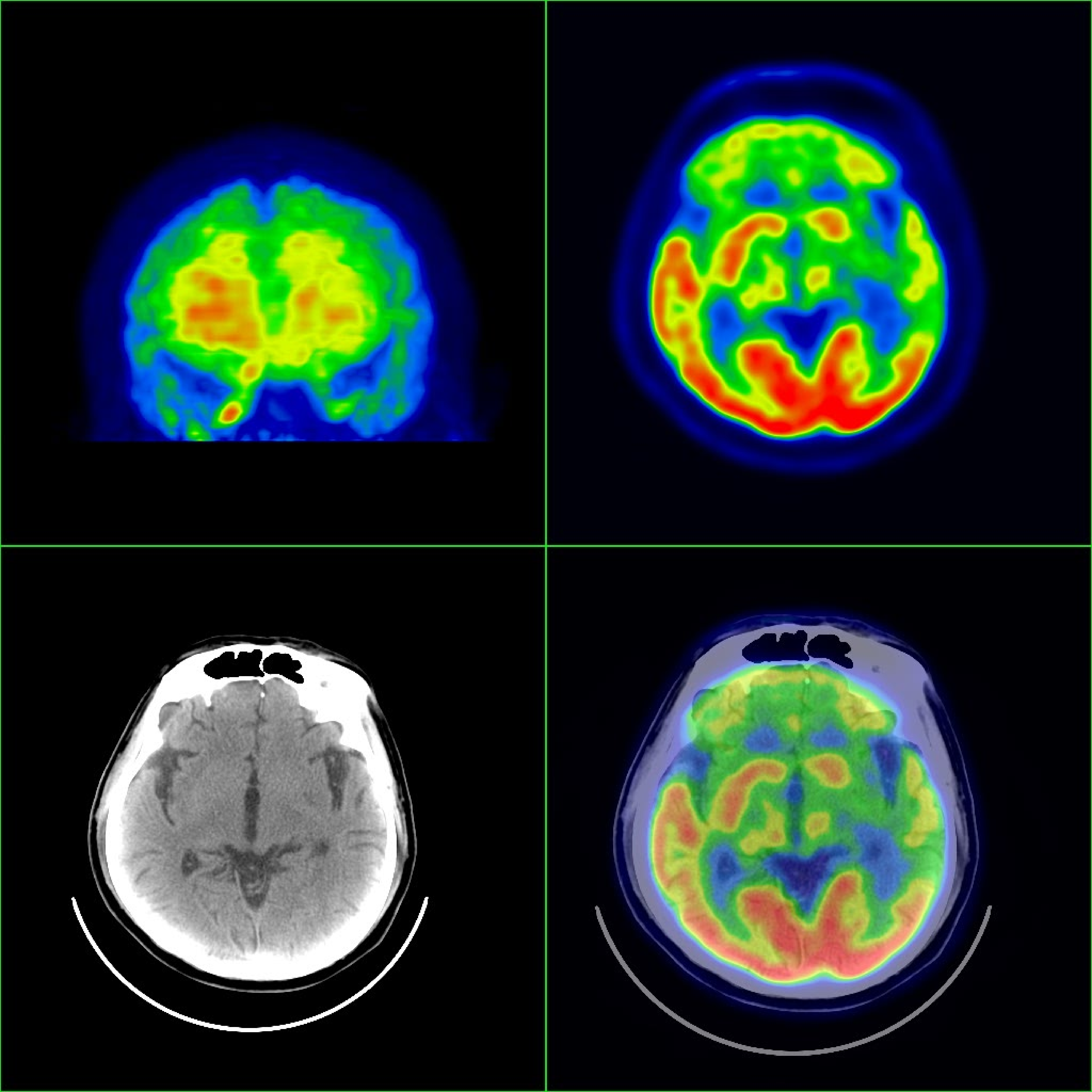

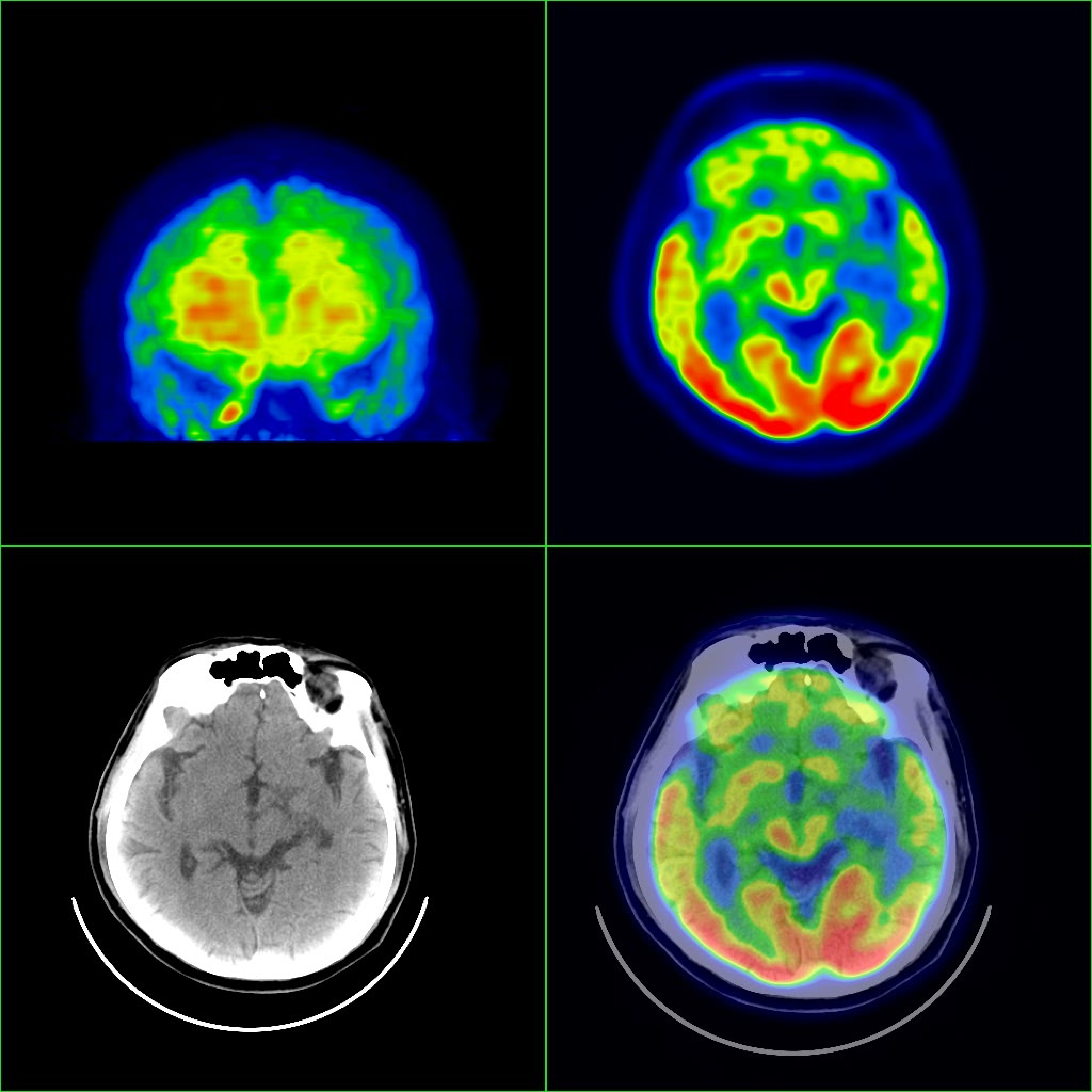

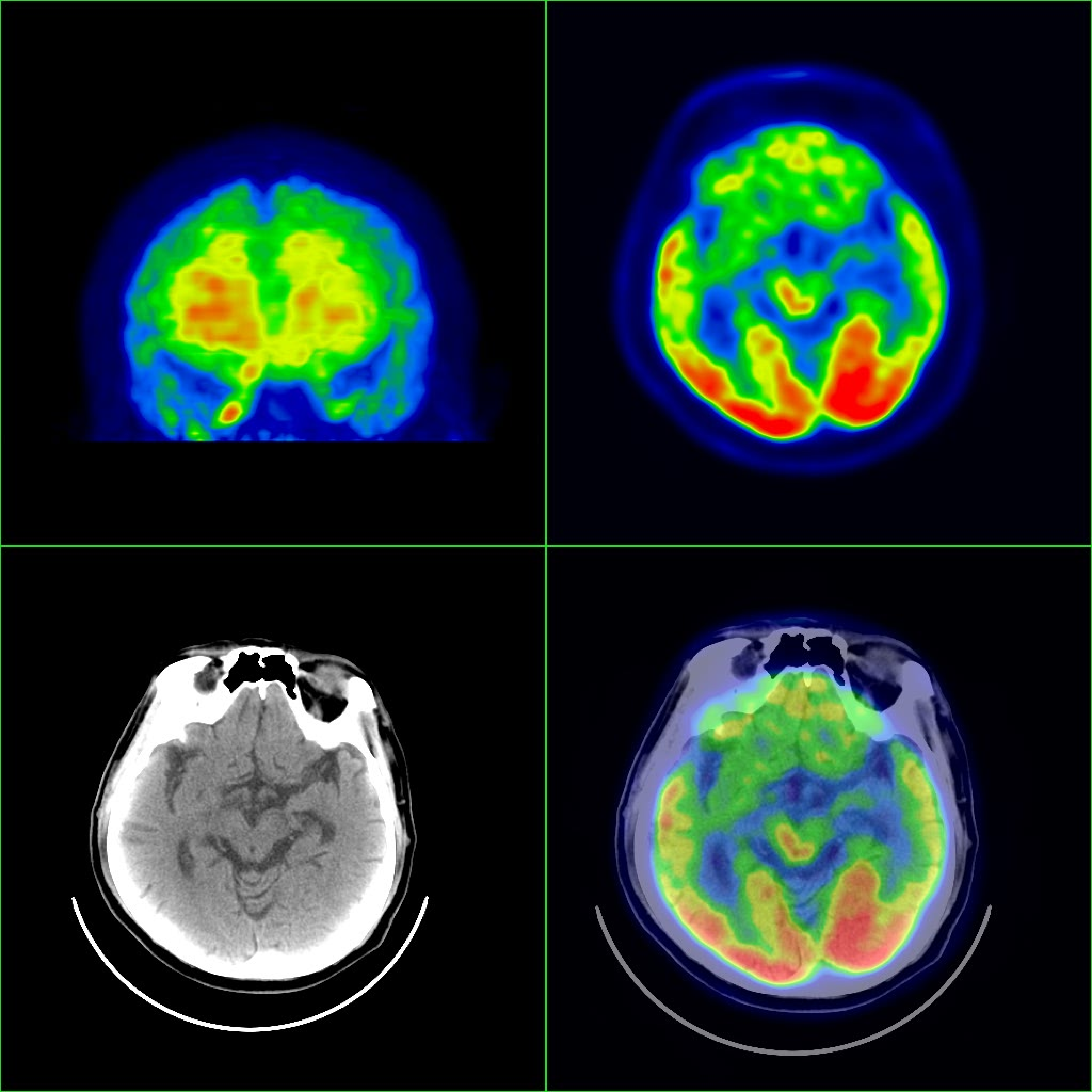

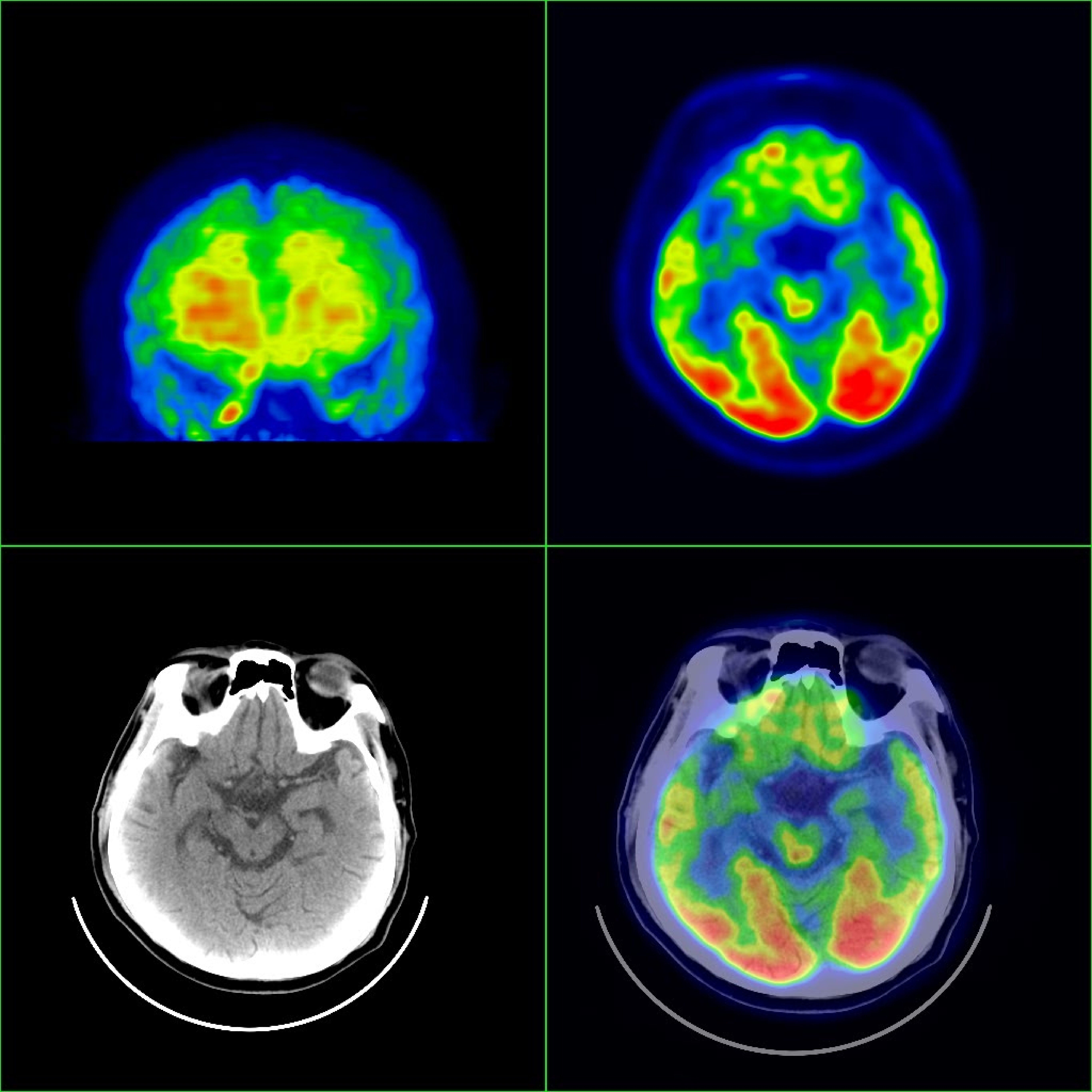

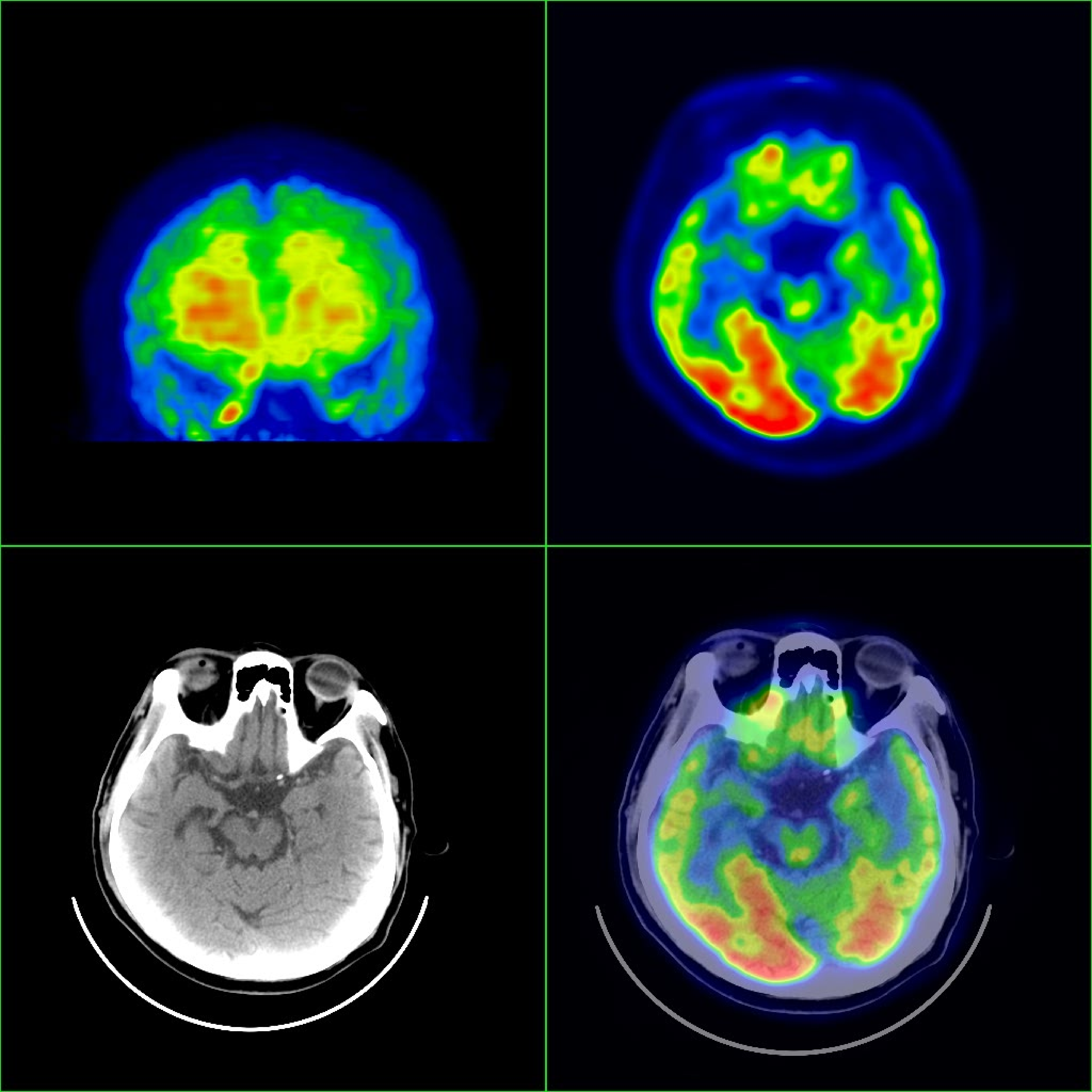

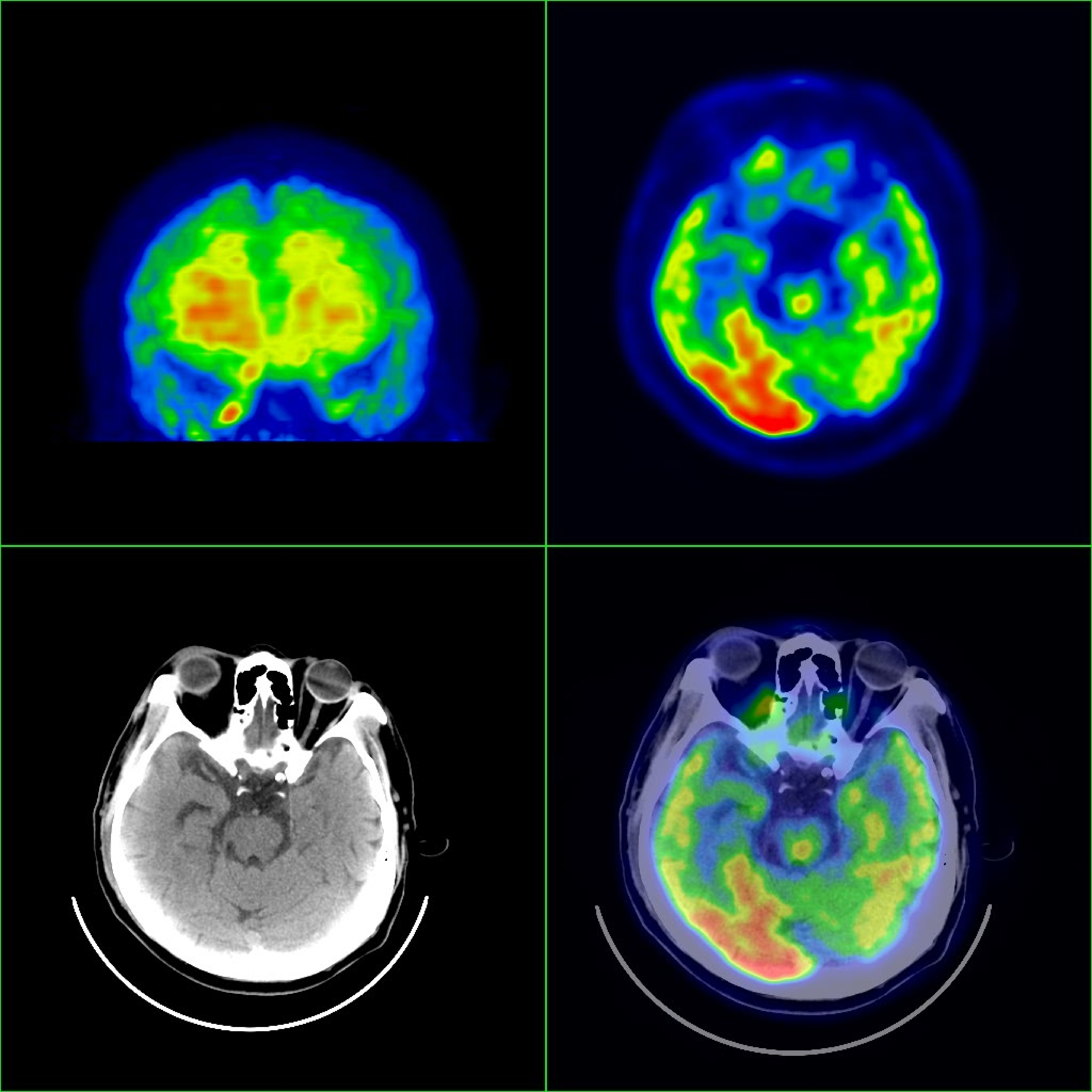

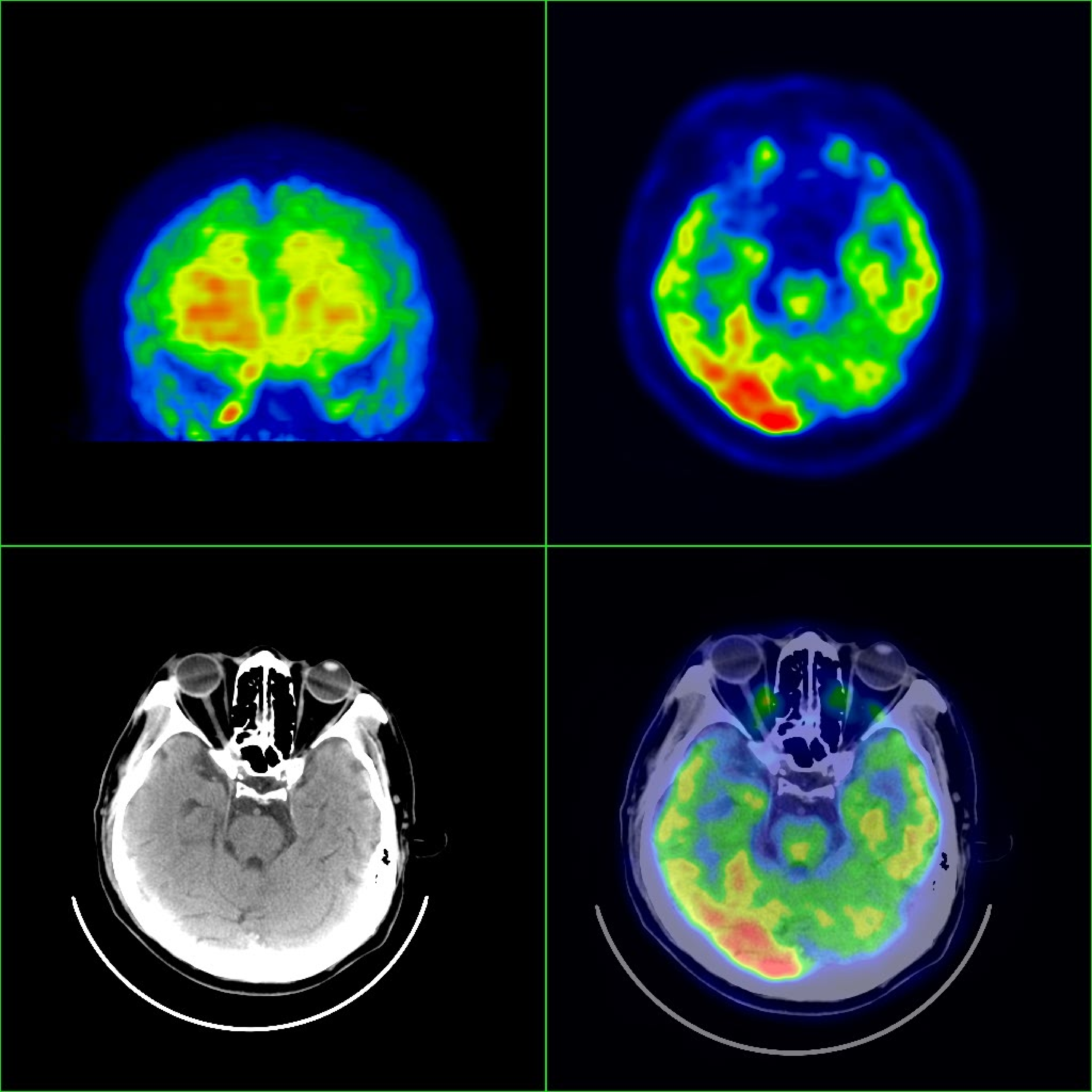

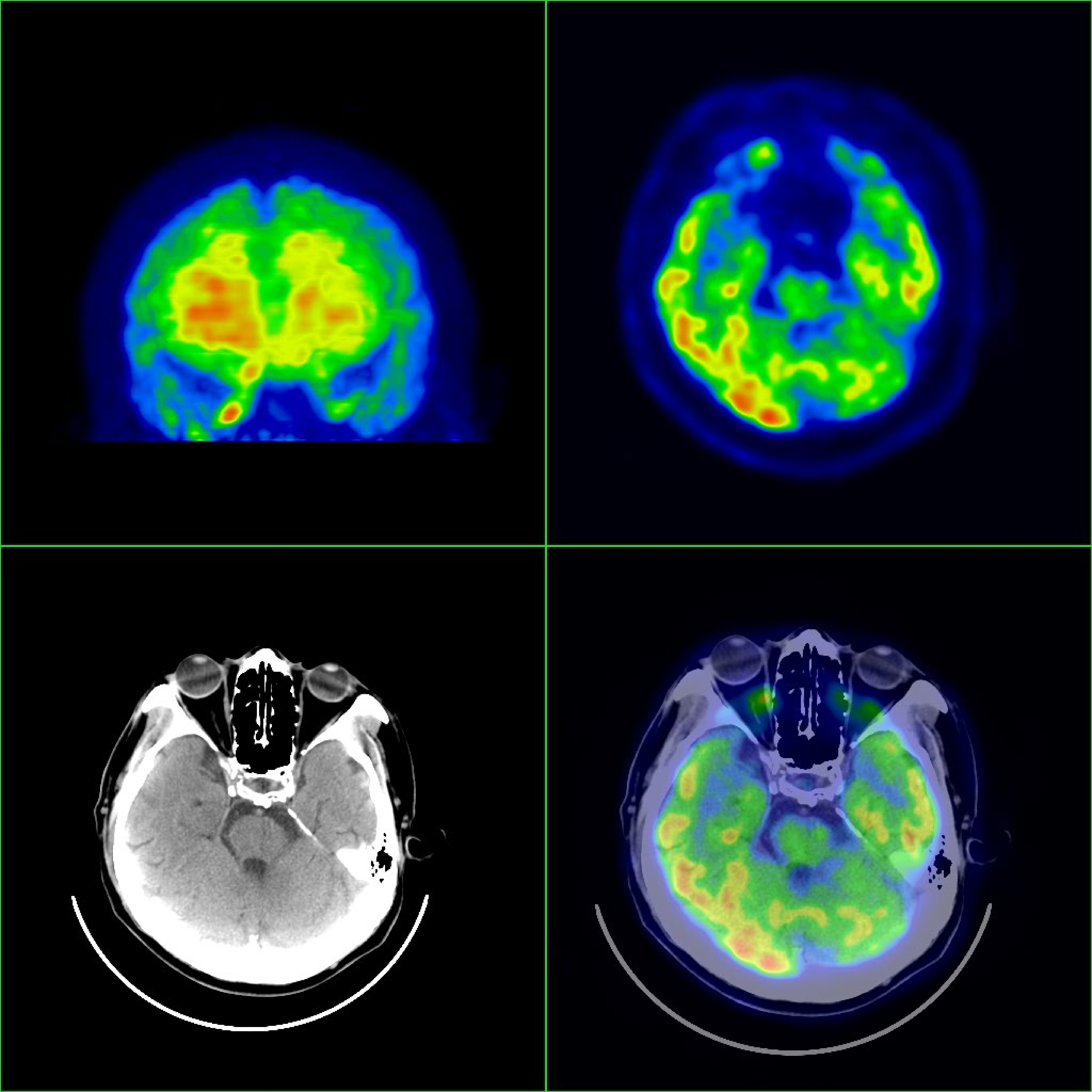

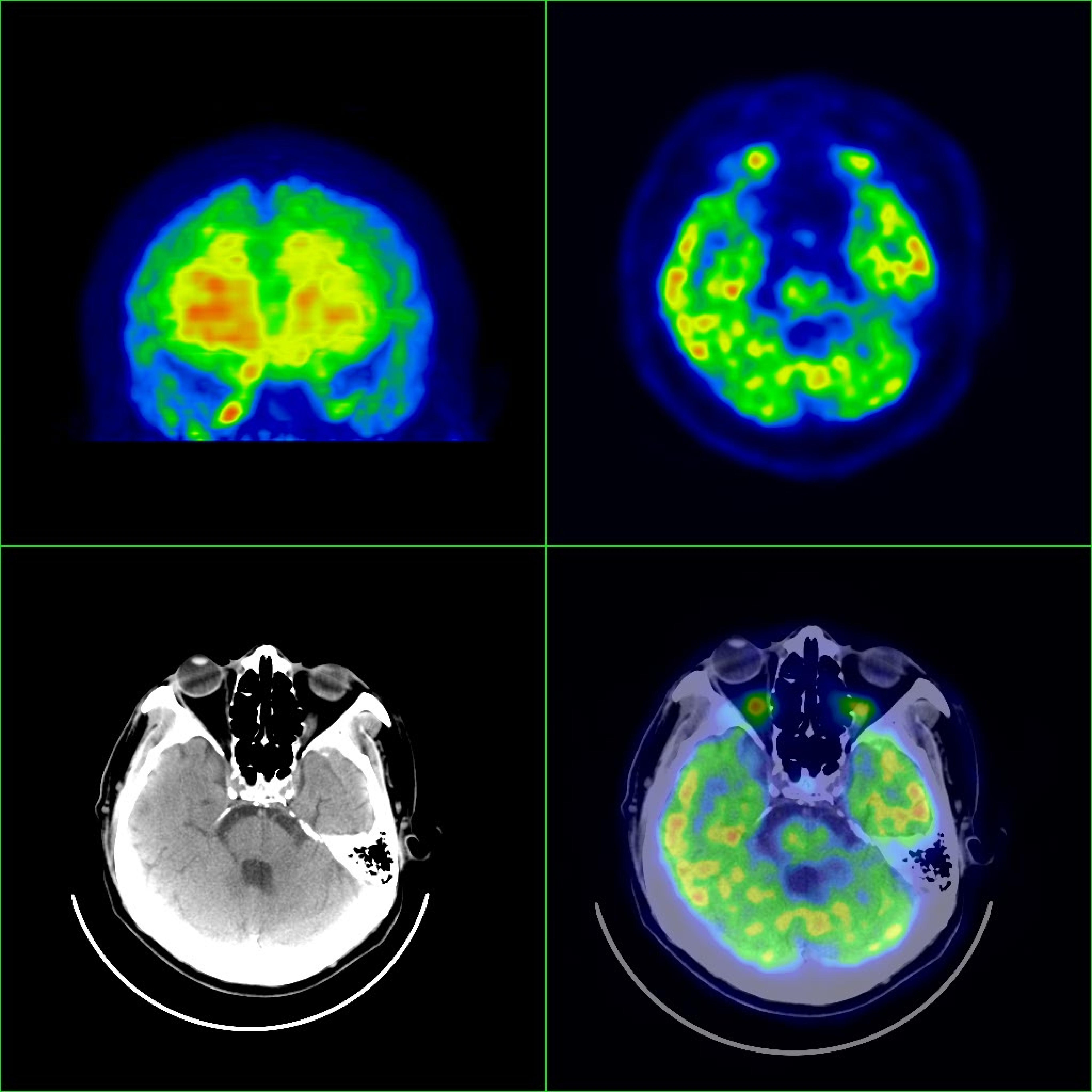

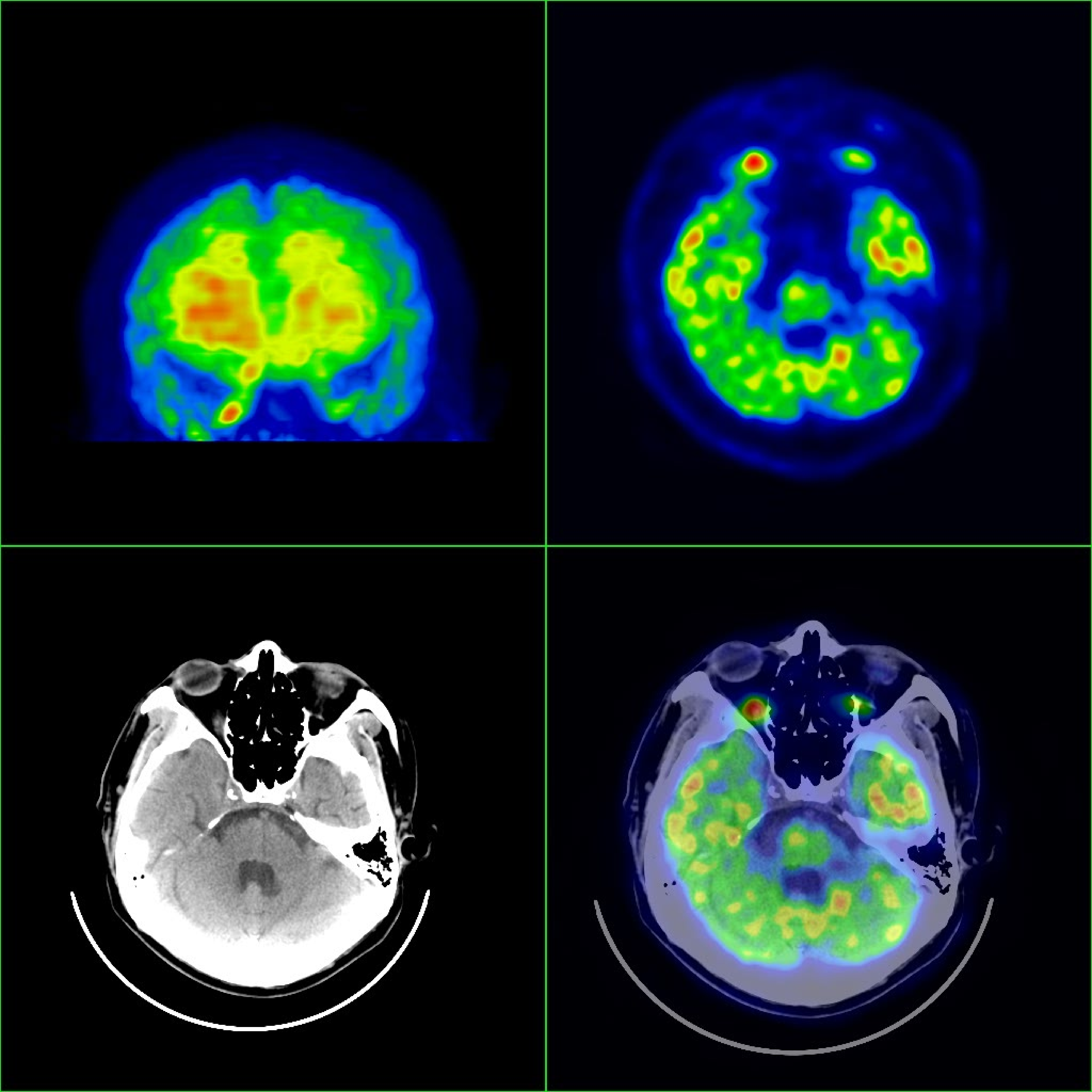

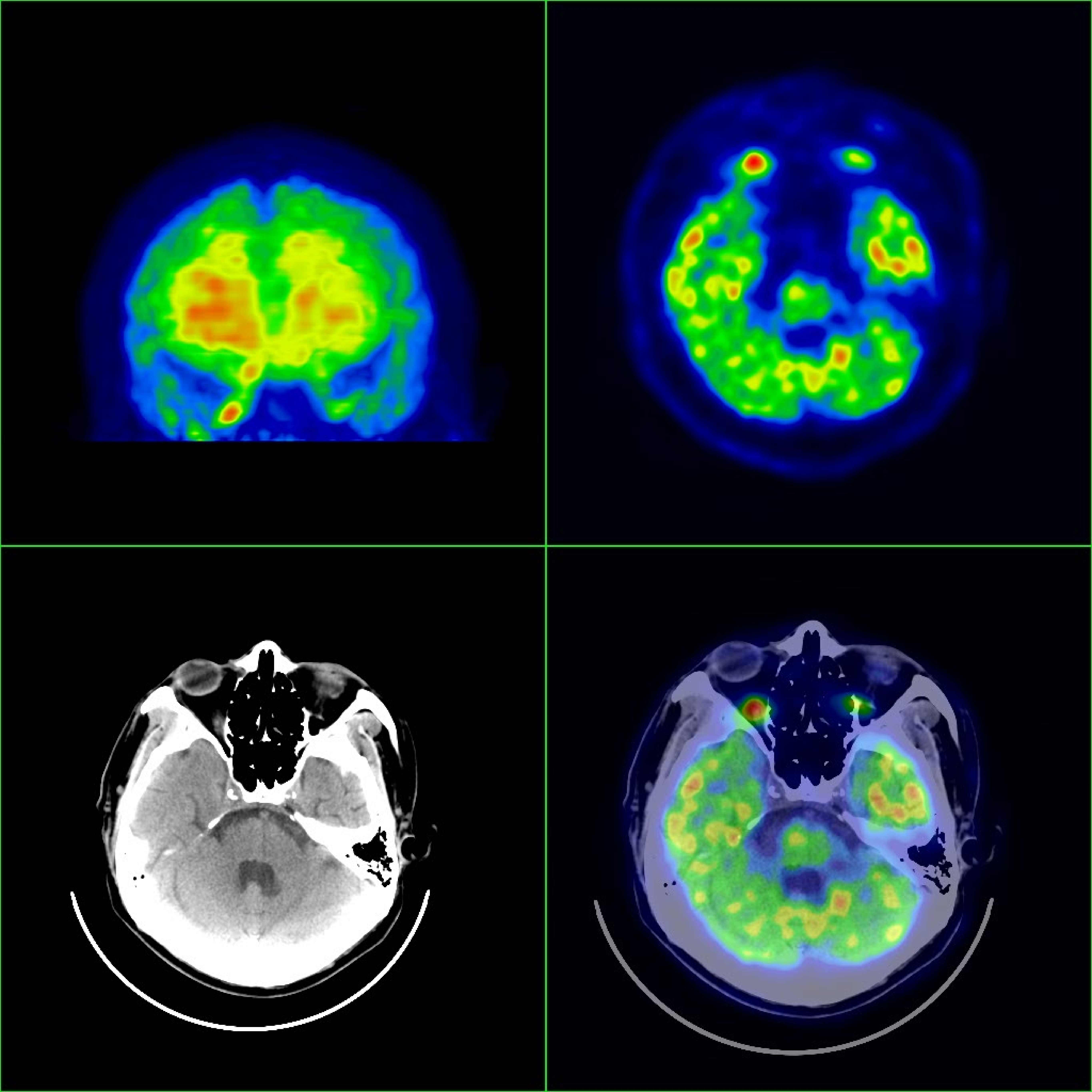

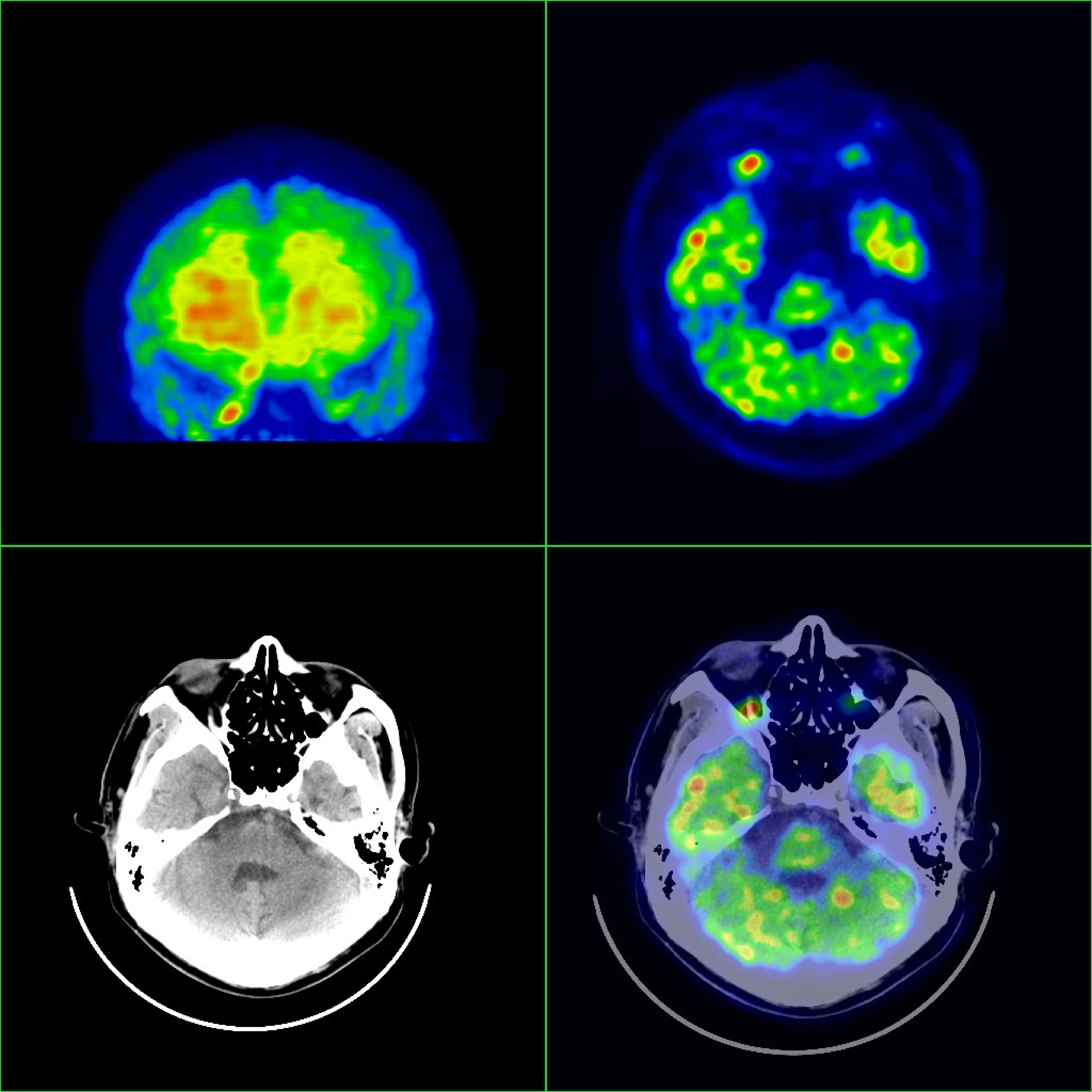

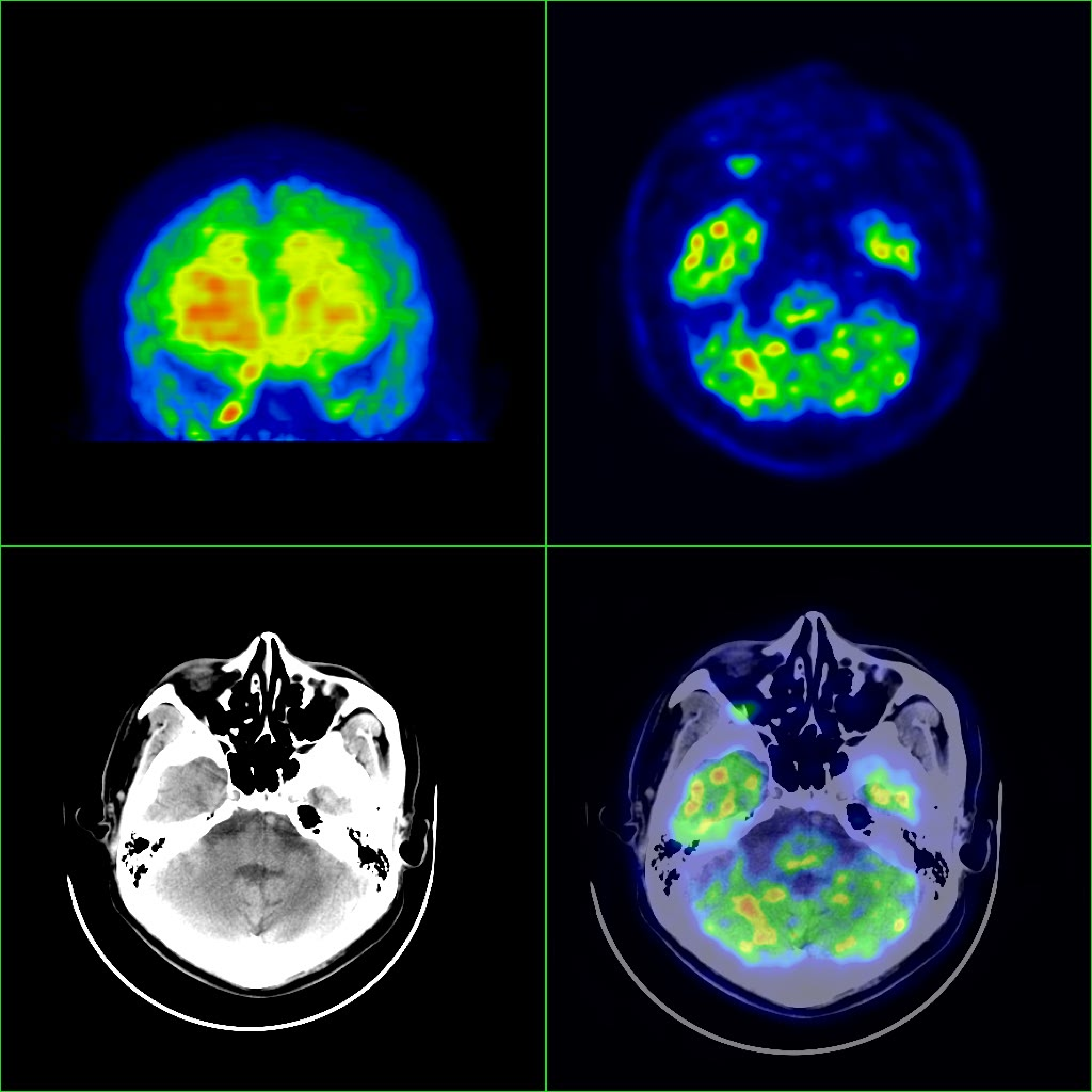

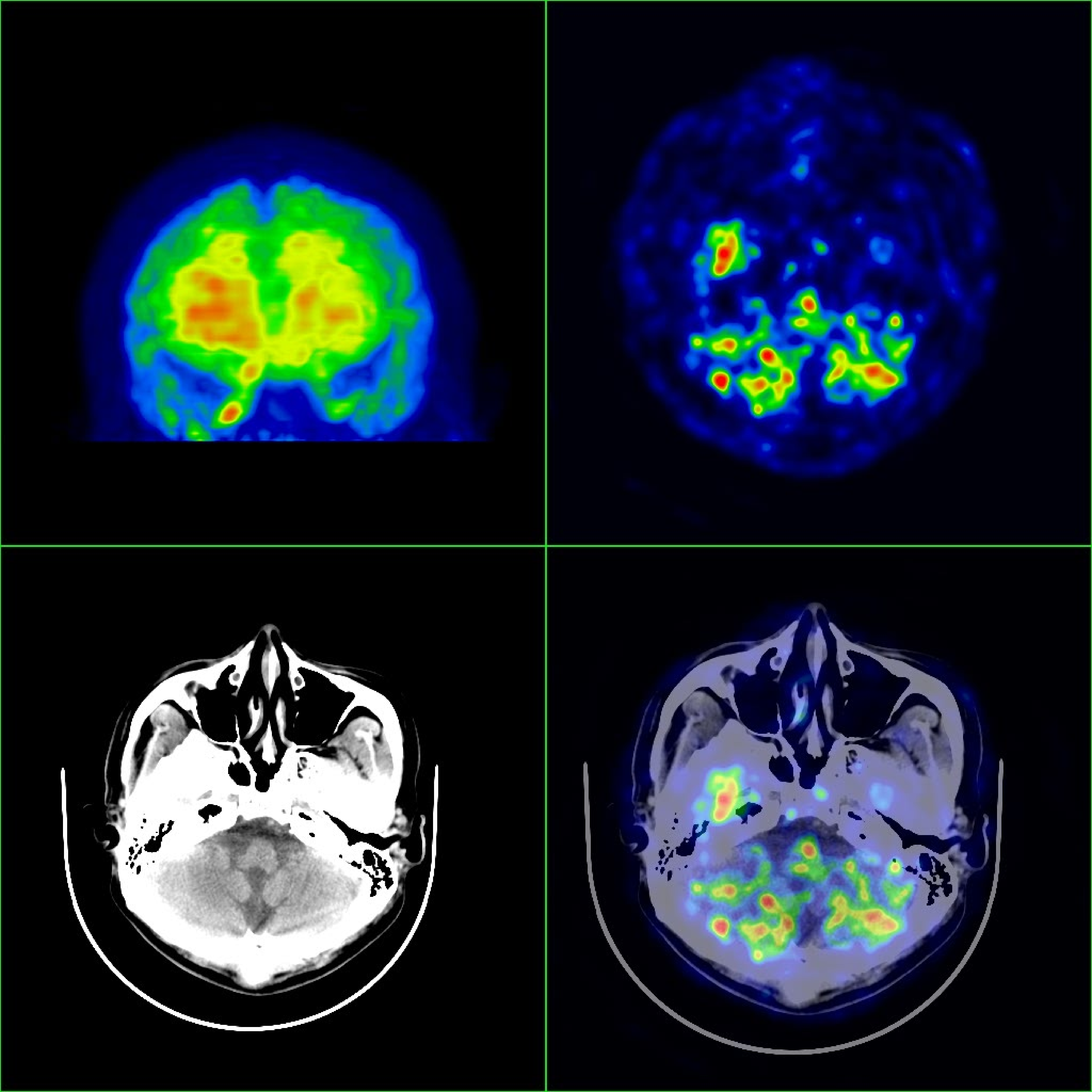

Supplement: Supplementary file 1 [file DataSheet_1.pdf]
