# Supplementary material for: Case report: Ofatumumab treatment in anti-DPPX autoimmune encephalitis
Source: Front Immunol. 2024 Jun 27;15:1320608. doi: 10.3389/fimmu.2024.1320608 (PMC11240285; doi:10.3389/fimmu.2024.1320608)
Supplement: Supplementary file 2 [file Presentation_1.ppt]

## Slide 1
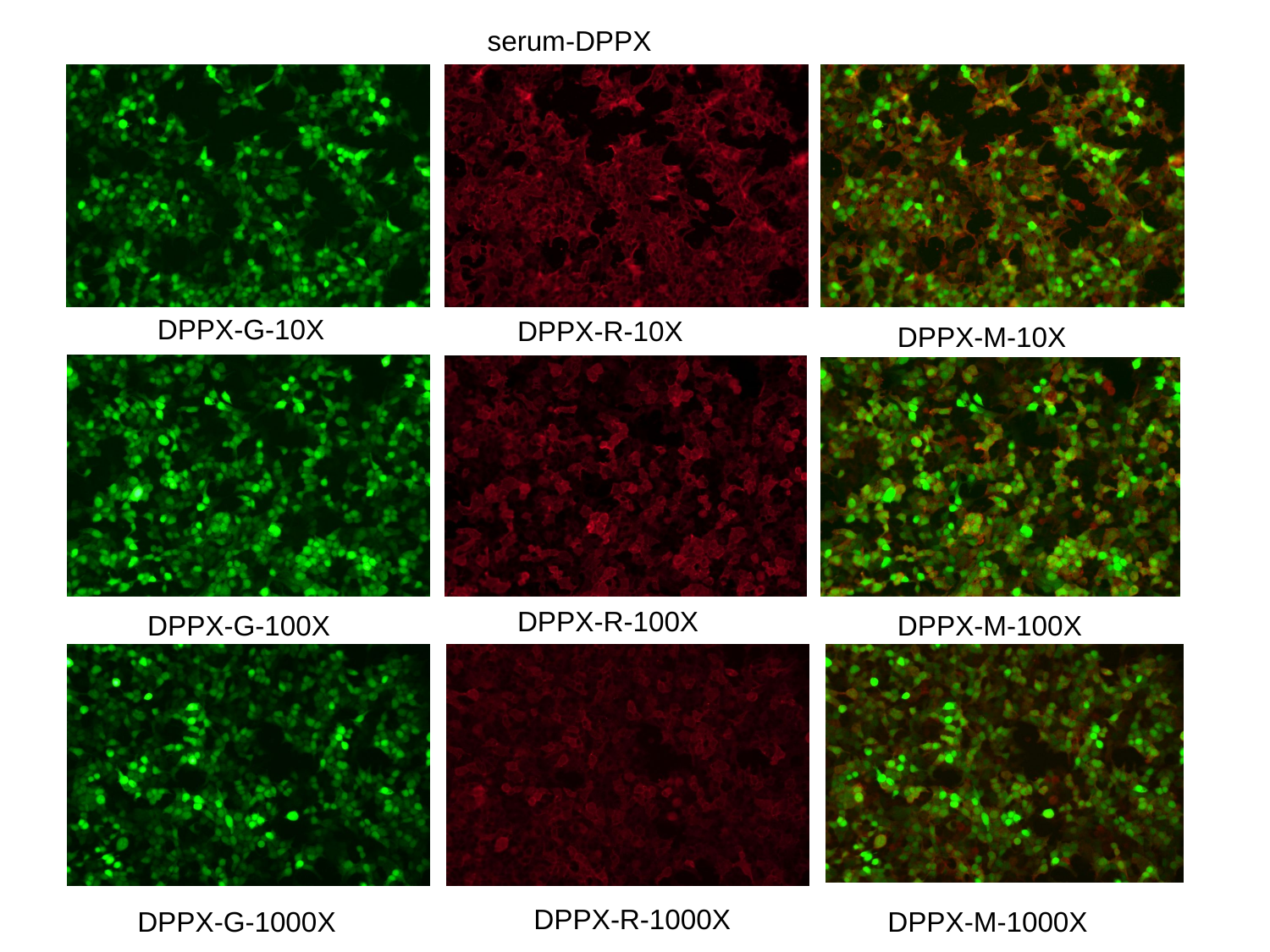

serum-DPPX
DPPX-G-10X
DPPX-R-10X
DPPX-M-10X
DPPX-R-100X
DPPX-G-100X
DPPX-M-100X
DPPX-R-1000X
DPPX-G-1000X
DPPX-M-1000X

## Slide 2
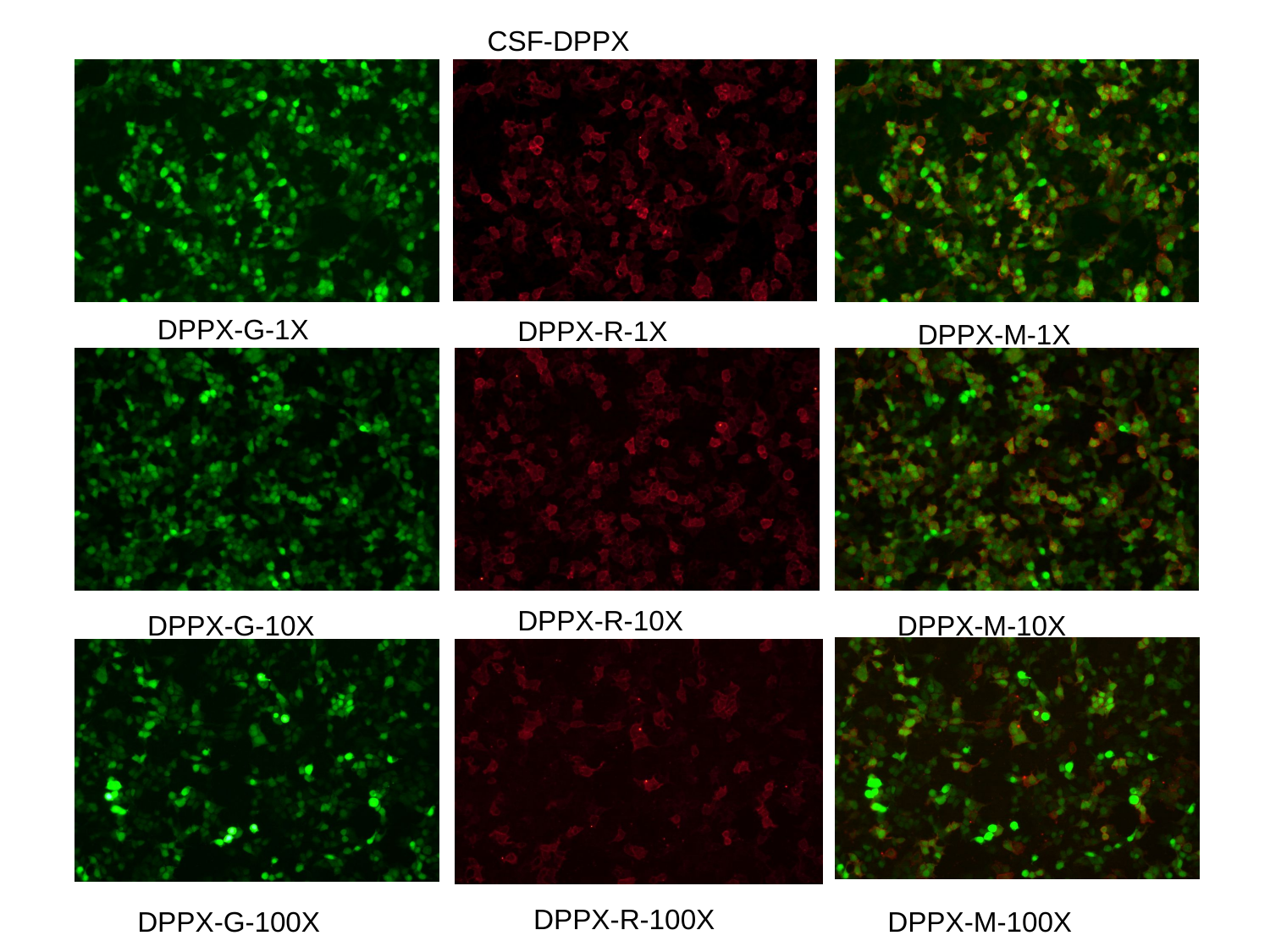

CSF-DPPX
DPPX-G-1X
DPPX-R-1X
DPPX-M-1X
DPPX-R-10X
DPPX-G-10X
DPPX-M-10X
DPPX-R-100X
DPPX-G-100X
DPPX-M-100X

## Slide 3
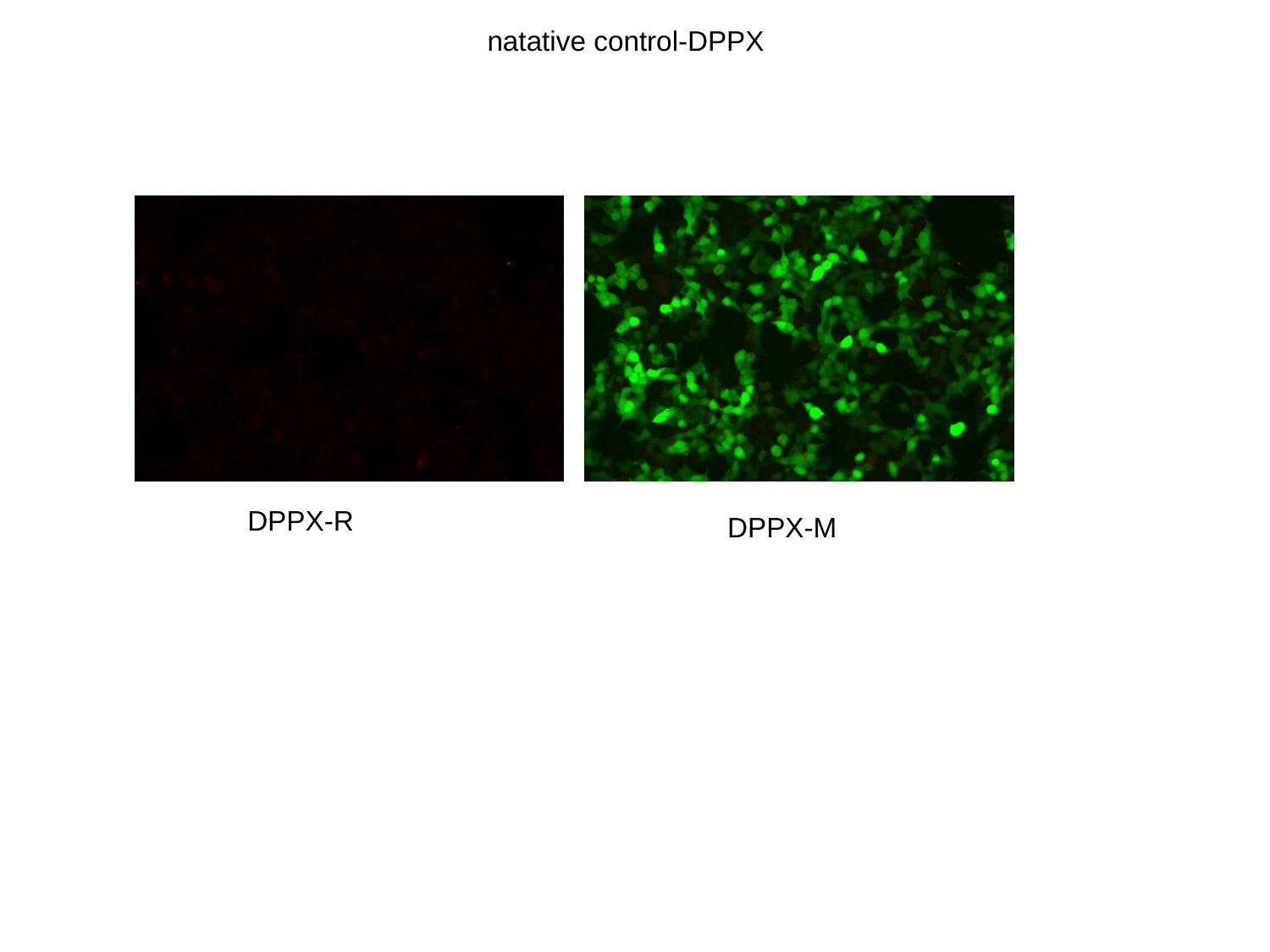

natative control-DPPX
DPPX-R
DPPX-M
