# Supplementary figures and images for: Case report: Ofatumumab treatment in anti-DPPX autoimmune encephalitis
Source: Front Immunol. 2024 Jun 27;15:1320608. doi: 10.3389/fimmu.2024.1320608 (PMC11240285; doi:10.3389/fimmu.2024.1320608)

## Slide 1
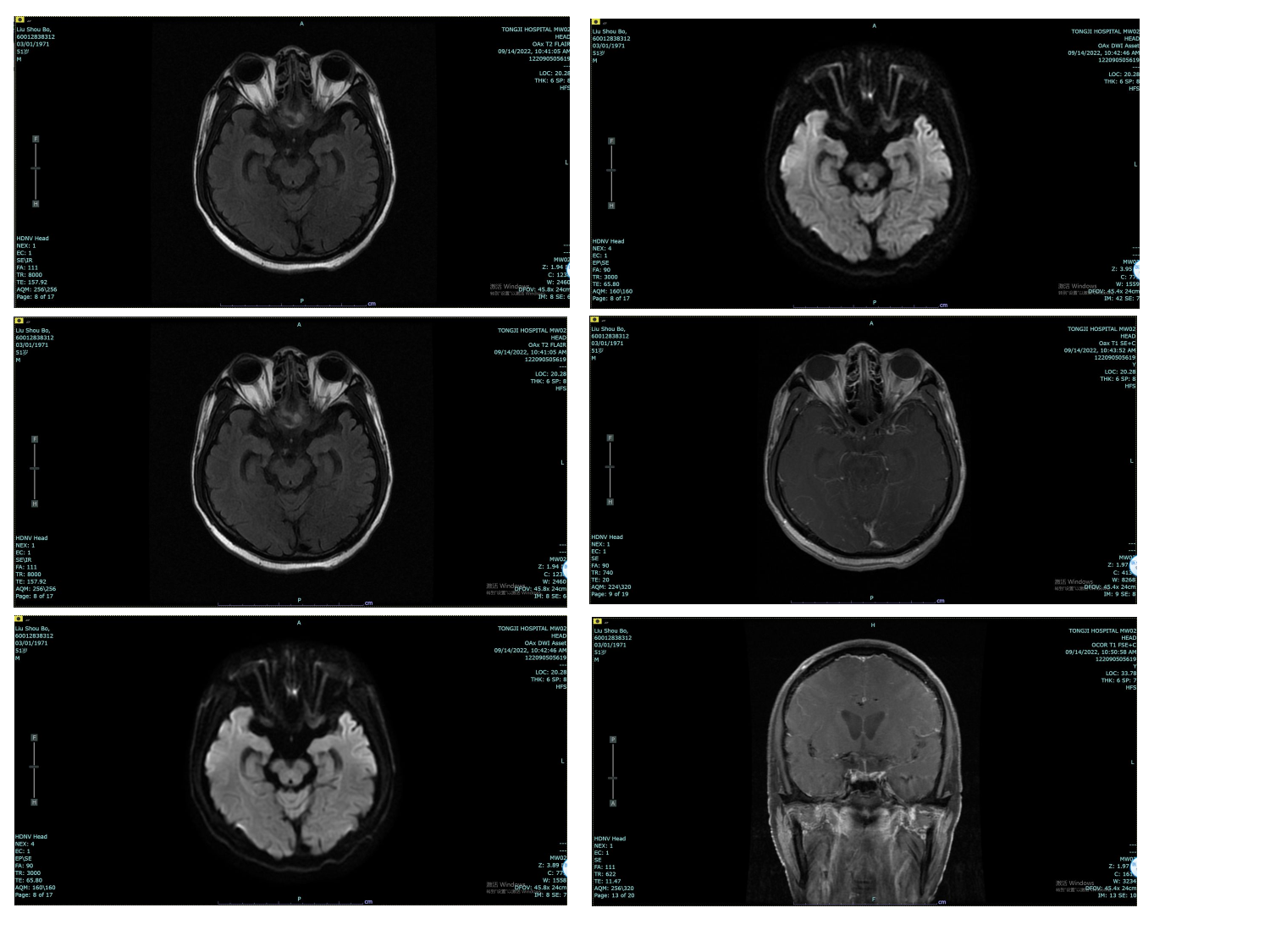

## Slide 2
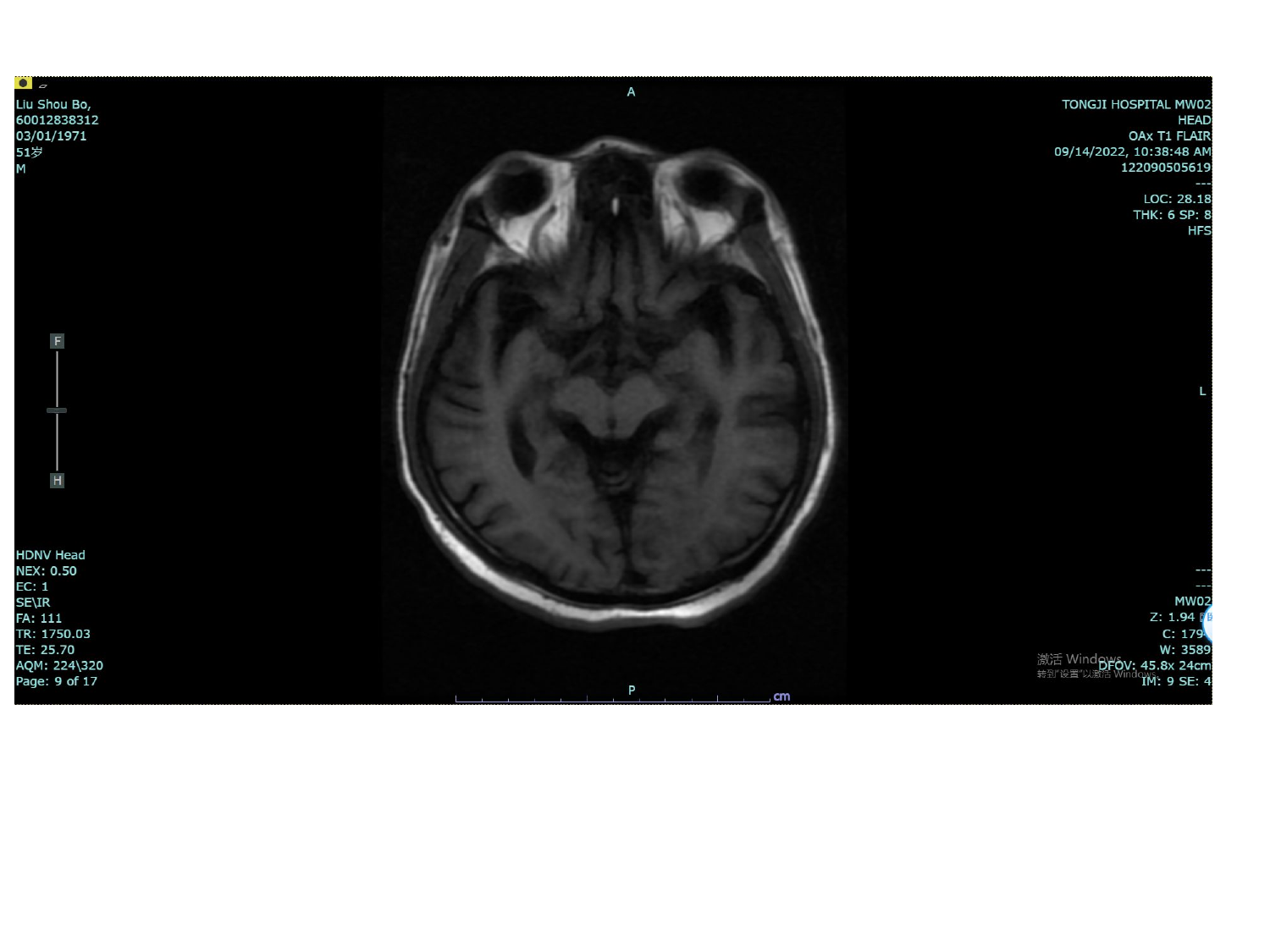

## Slide 3
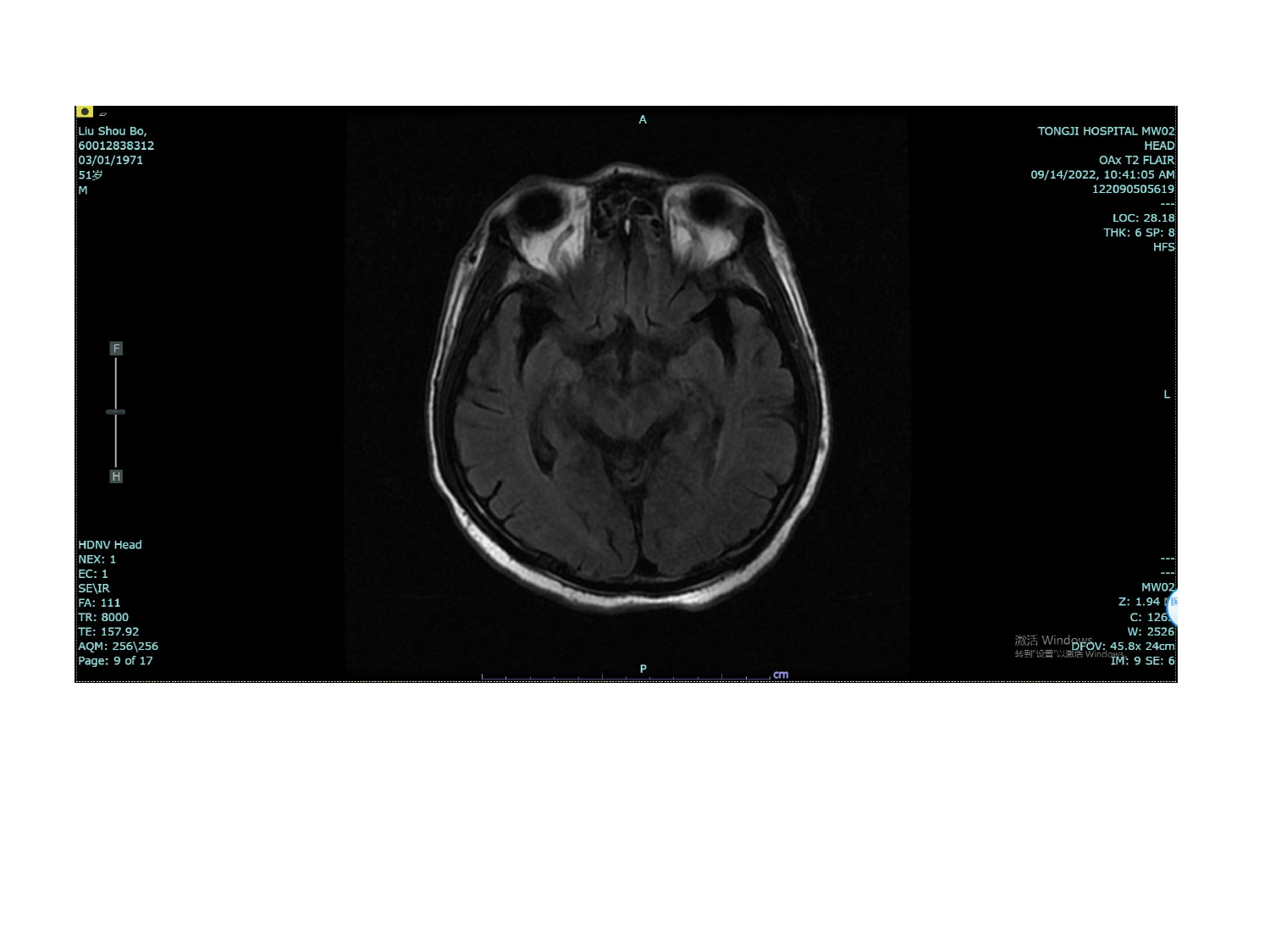

## Slide 4
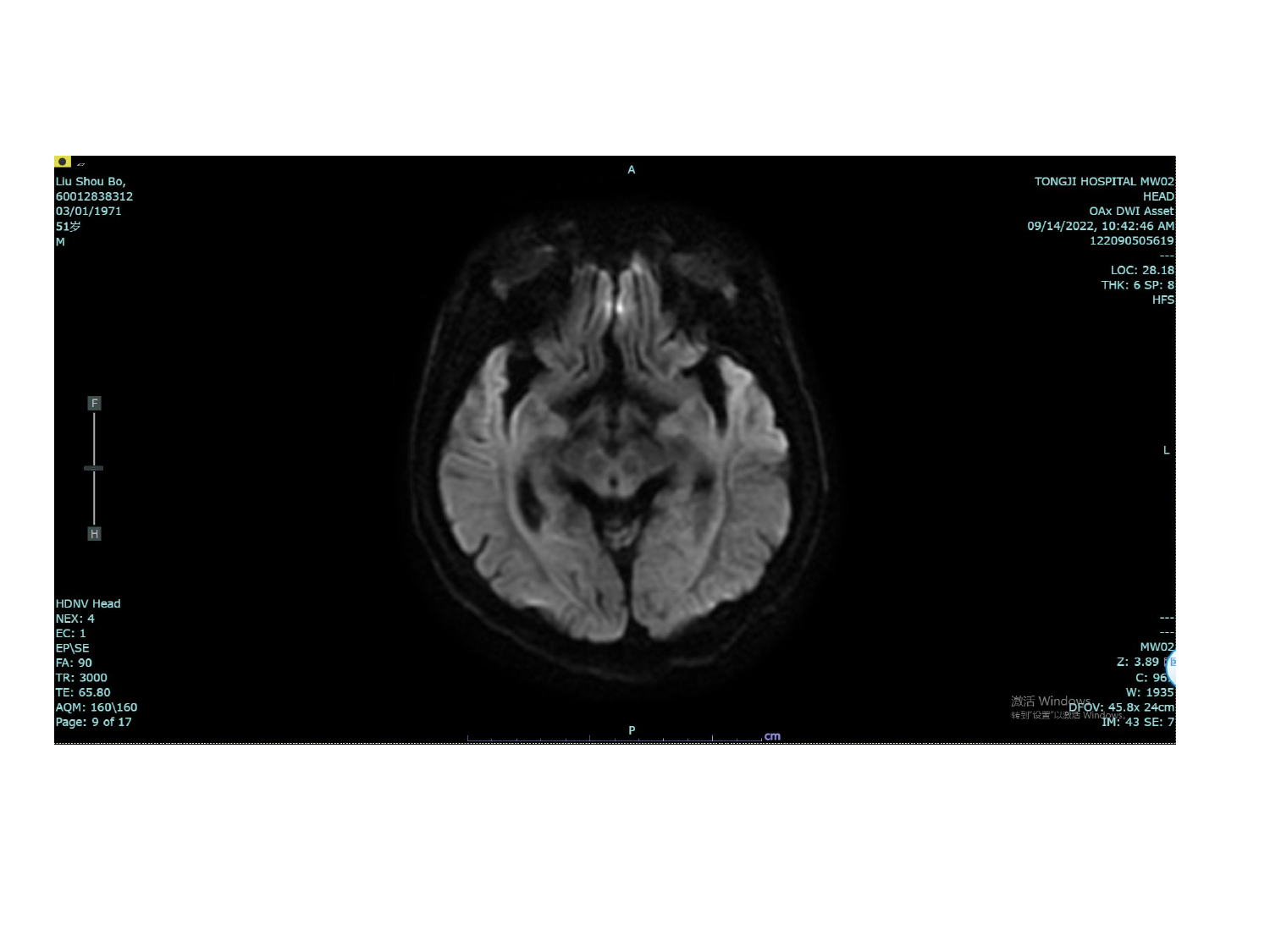

## Slide 5
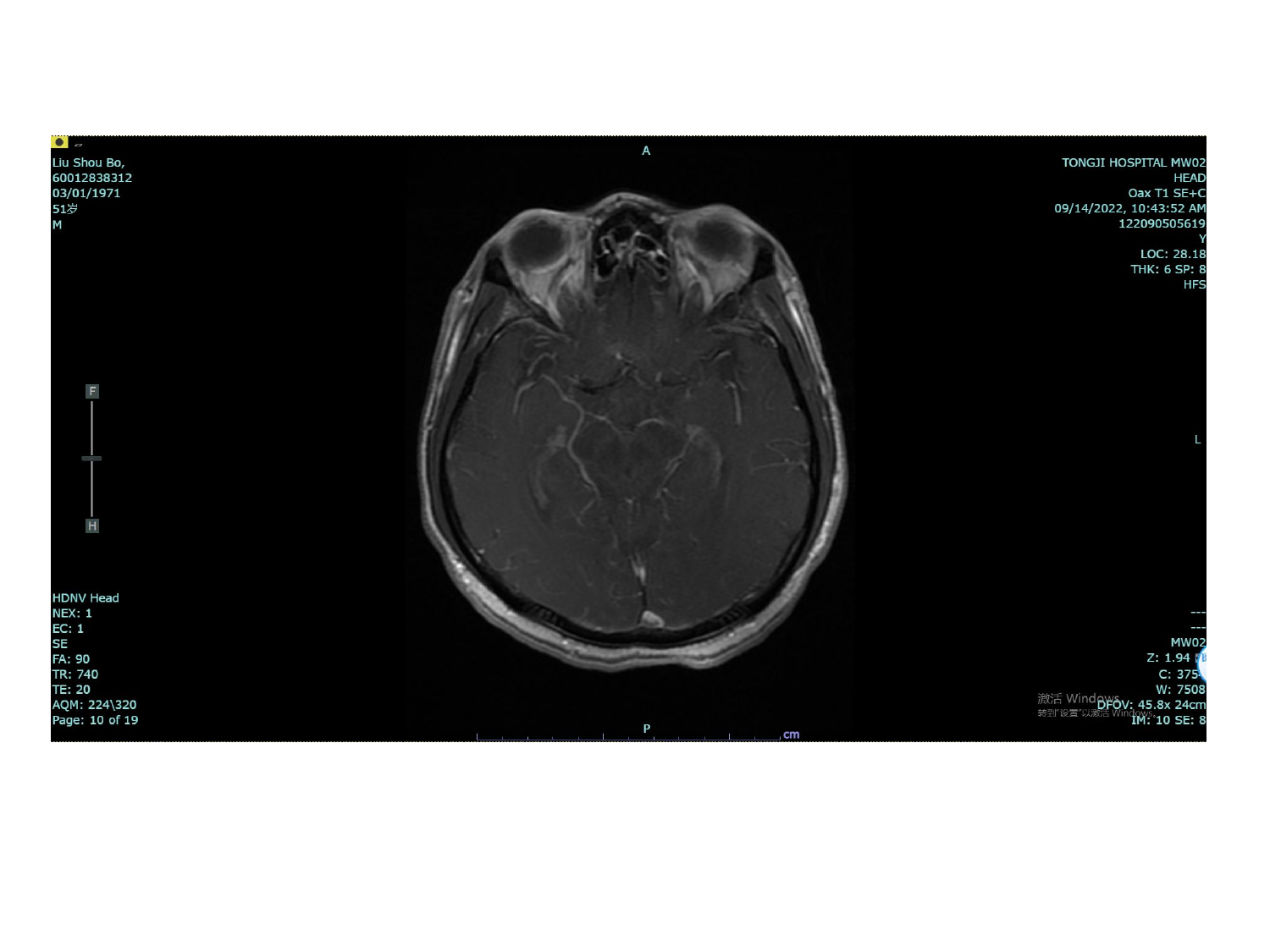

## Slide 6
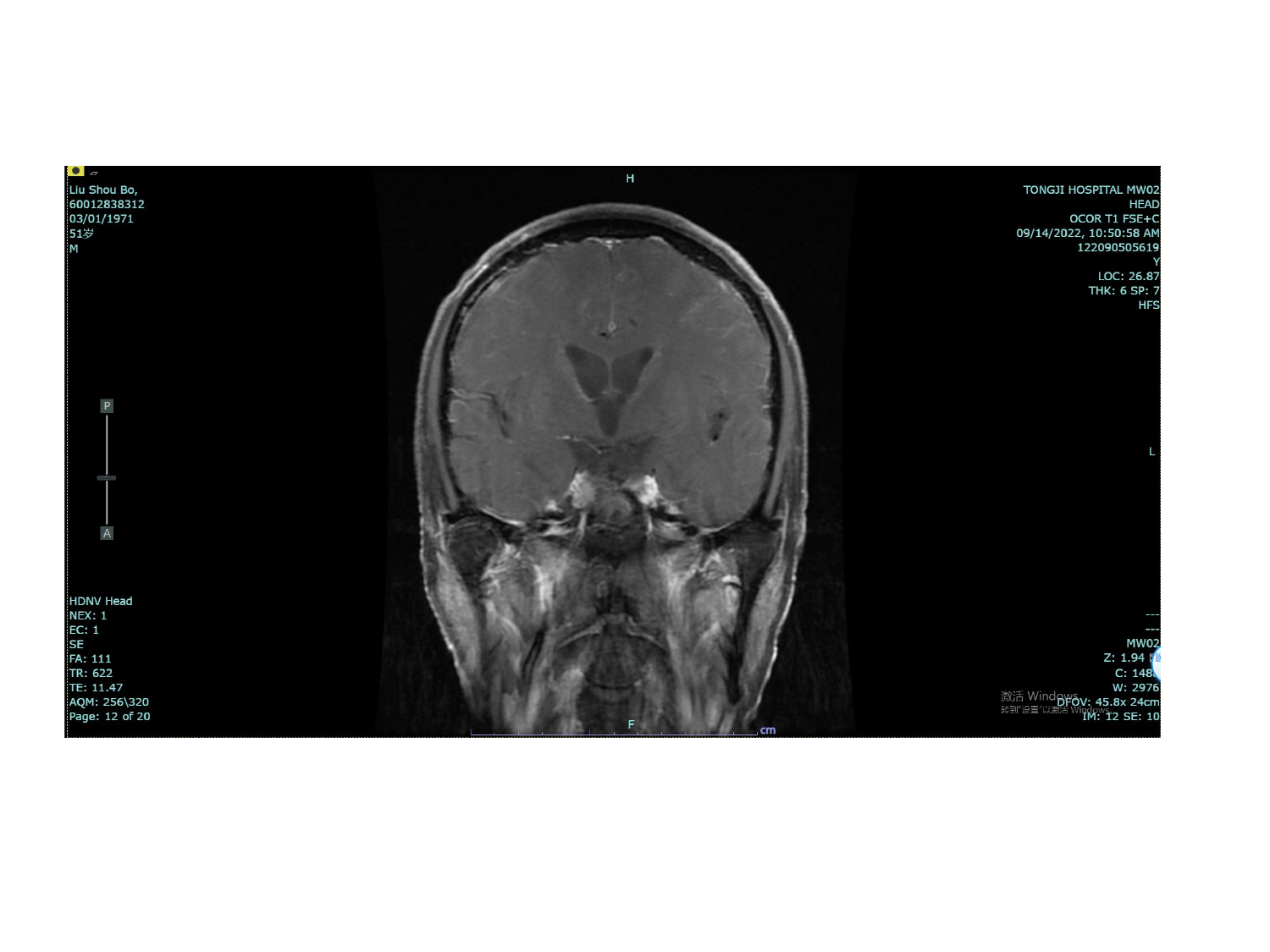

## Slide 7
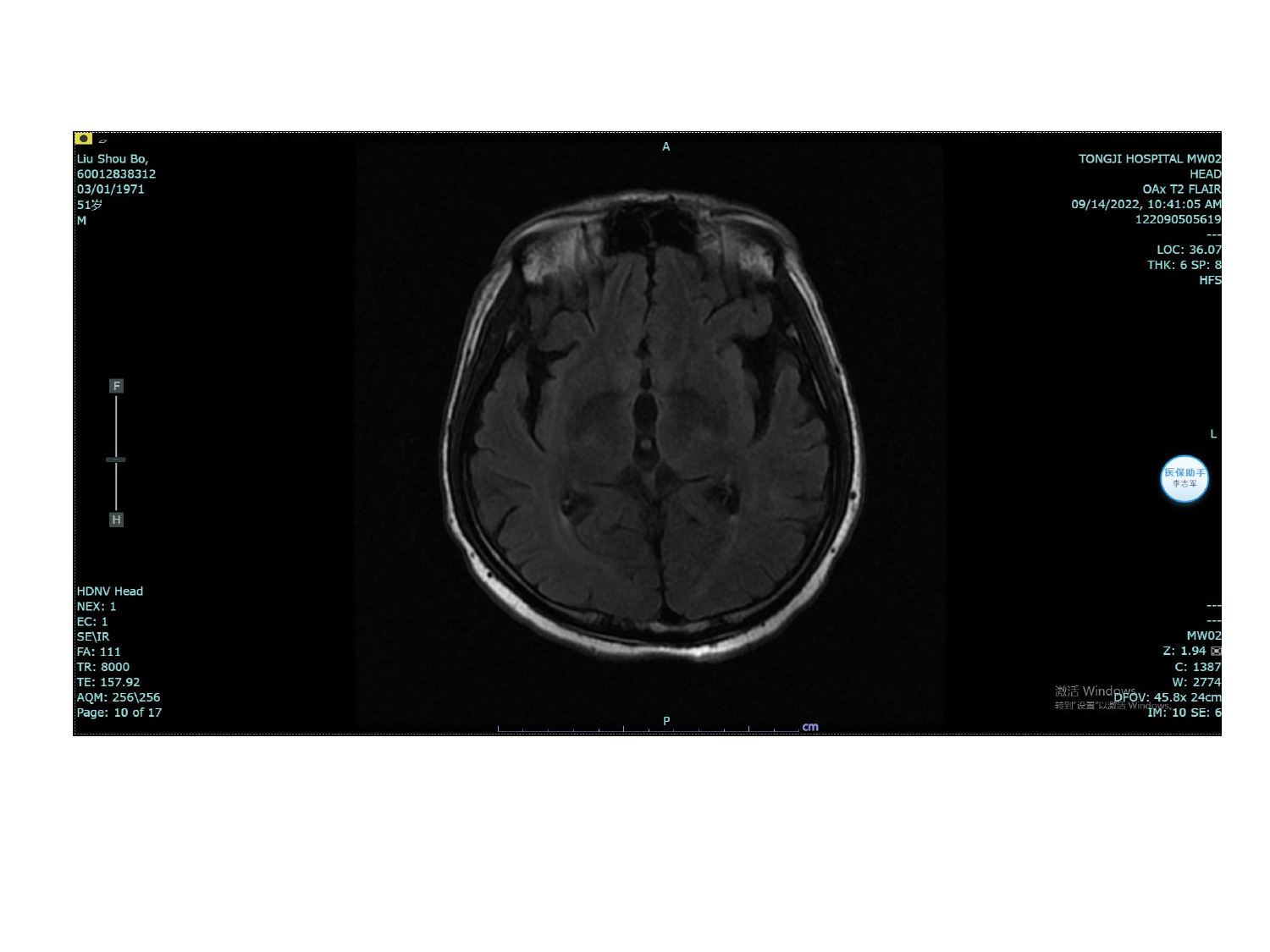

## Slide 8
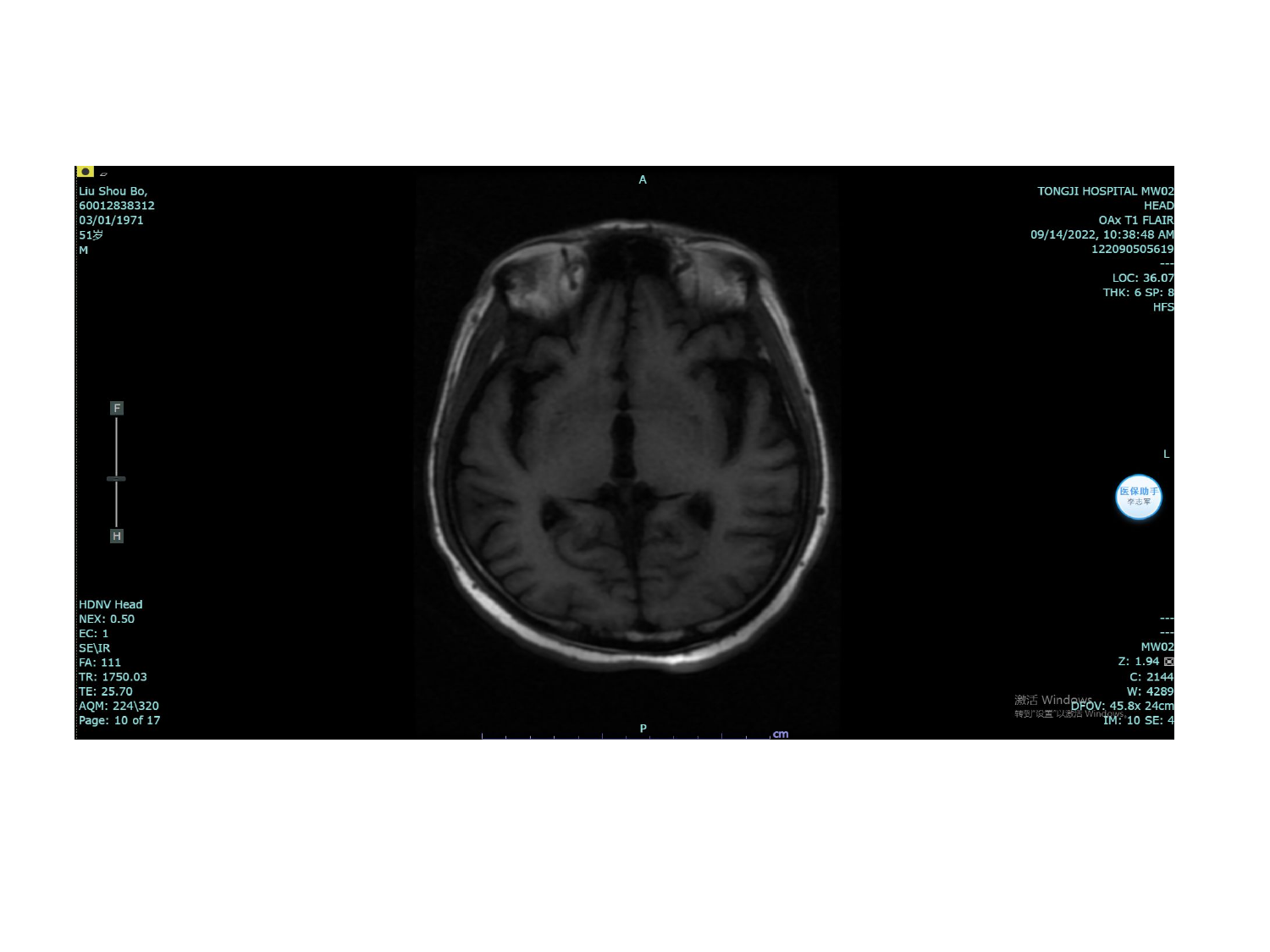

## Slide 9
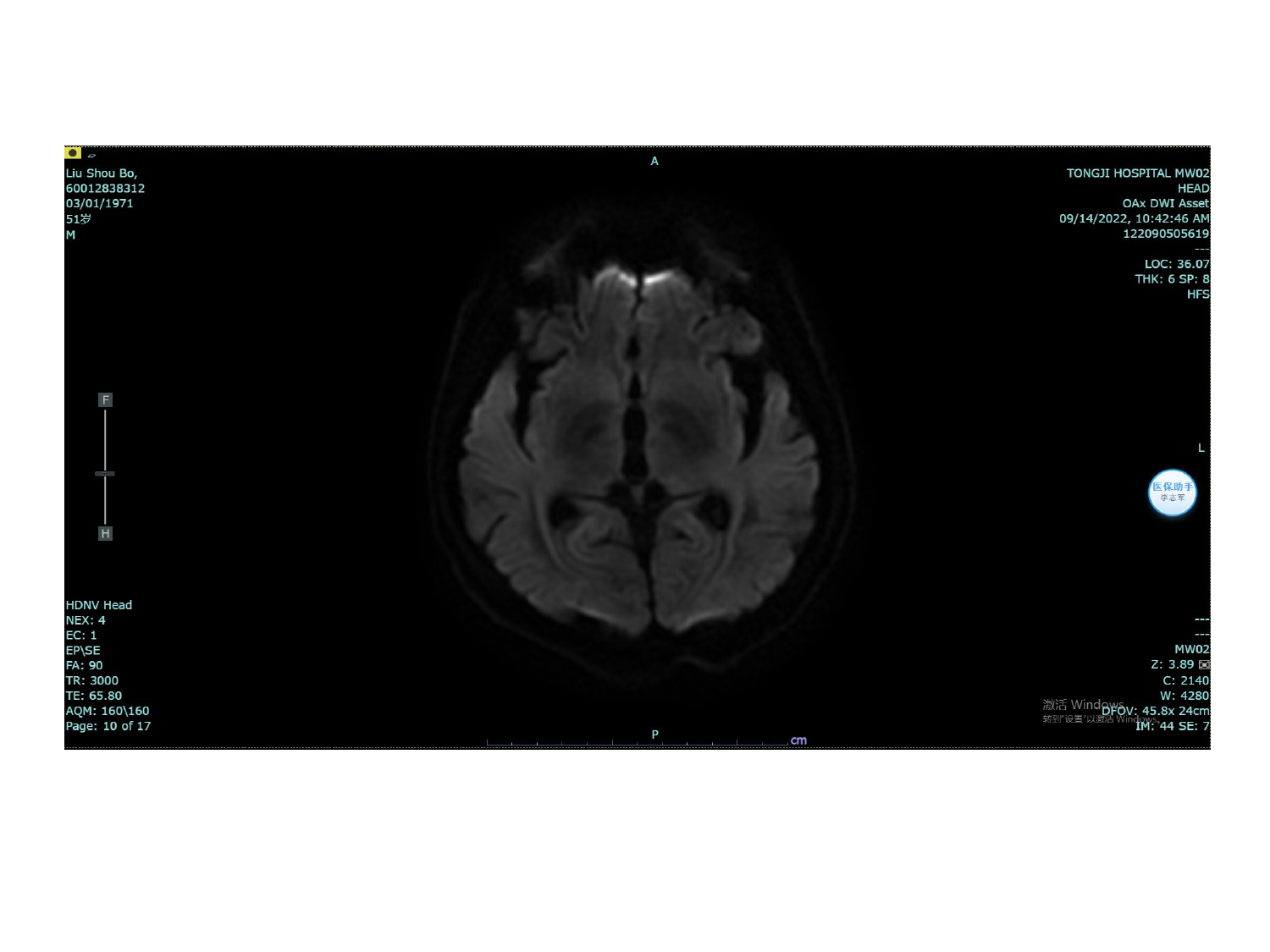

## Slide 10
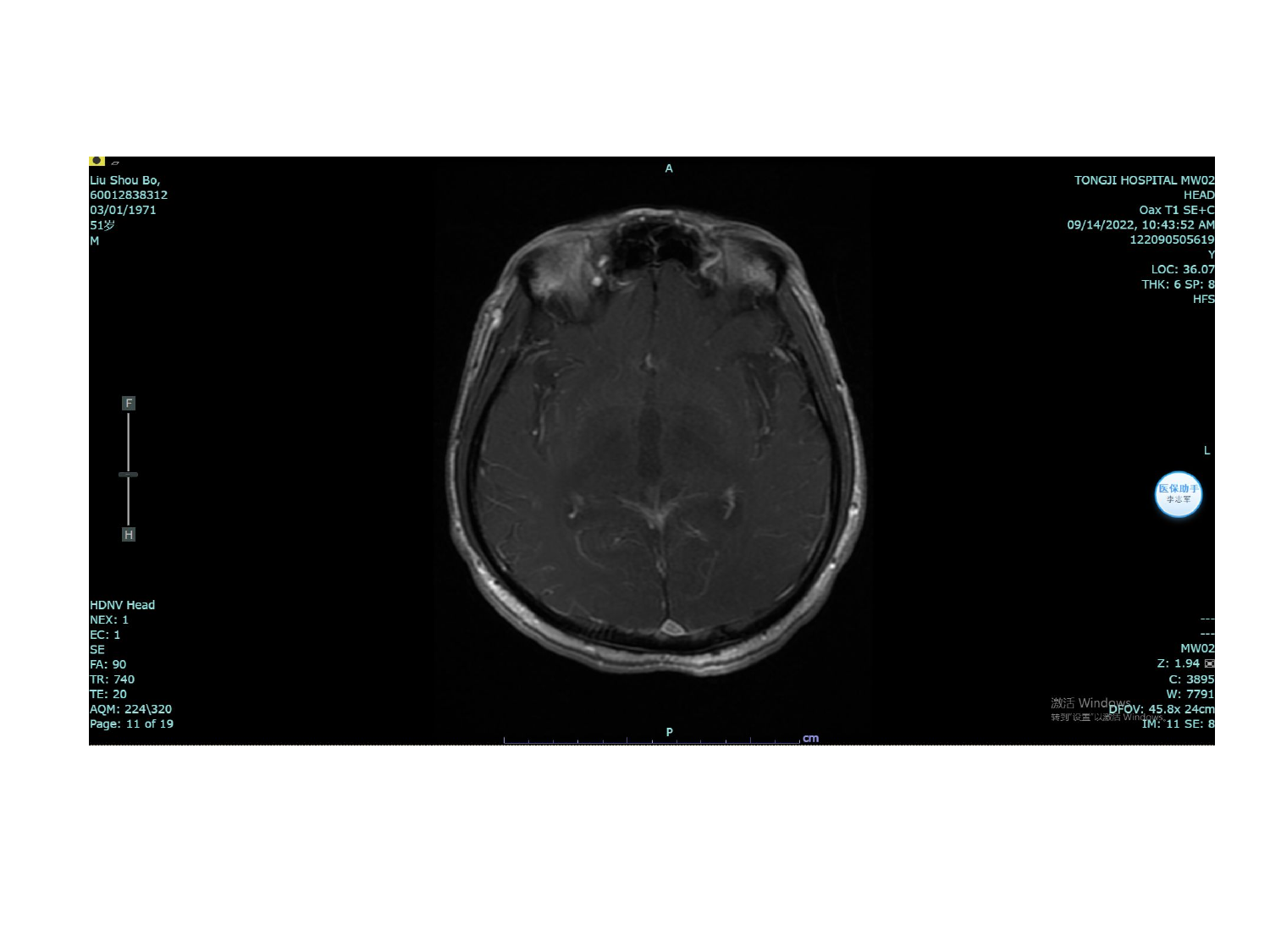

## Slide 11
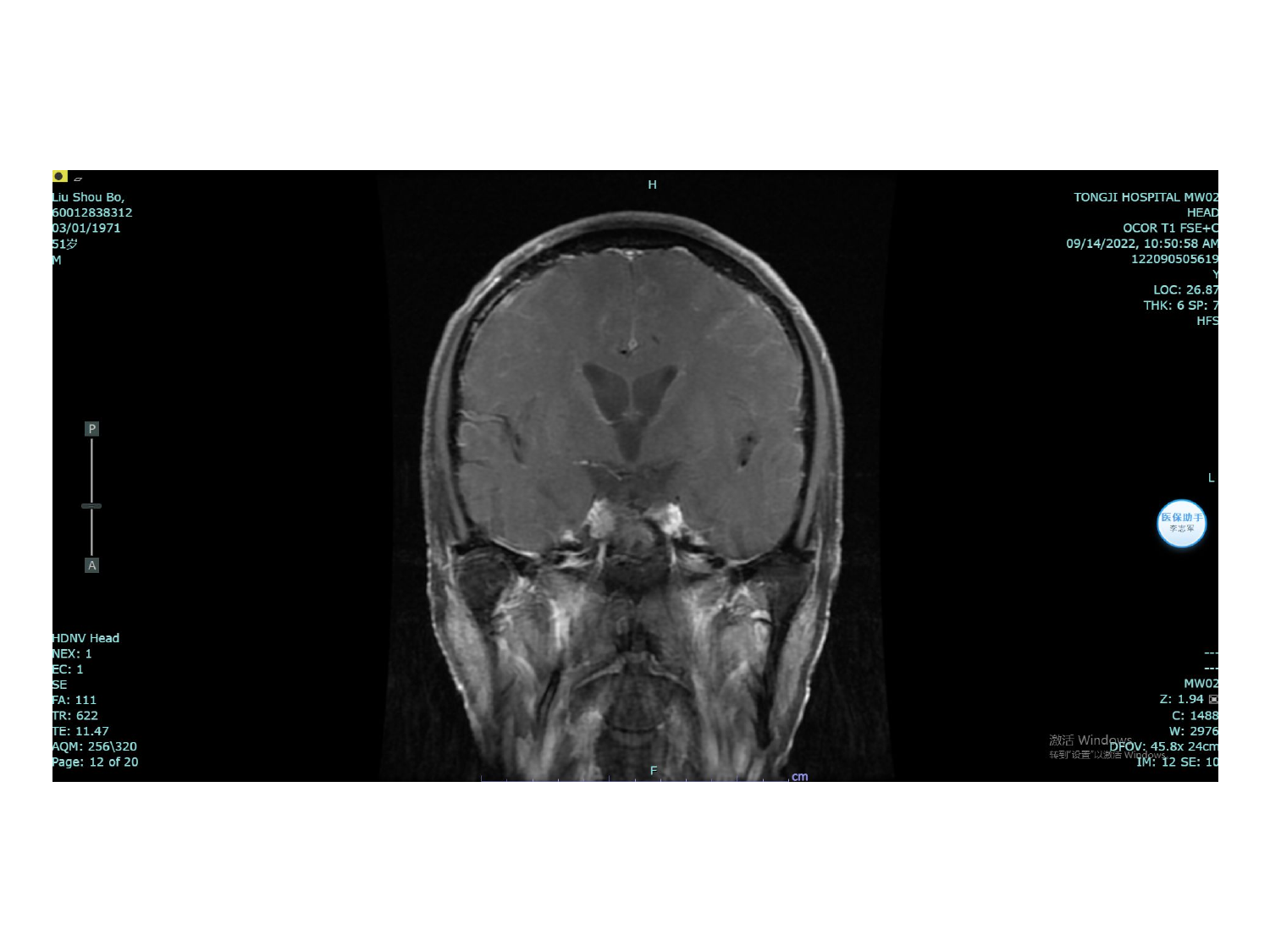

Supplement: Supplementary file 3 [file Presentation_2.ppt]
